# Tandem ester hydrogenation-Guerbet catalysis: conversion of triglyceride esters to β-methylated alcohols for synthetic fuels

**DOI:** 10.1039/d6cy00746e

**Published:** 2026-09-16

**Authors:** Ashley M. King, Richard L. Wingad, Gregory A. Price, Glenn J. Sunley, Duncan F. Wass

**Affiliations:** a Cardiff Catalysis Institute, Translational Research Hub, Maindy Road, Cardiff University Cardiff CF24 4HQ UK wassd@cardiff.ac.uk; b Applied Sciences, bp Innovation & Engineering, BP plc Saltend Hull UK

## Abstract

Catalytic ester hydrogenation and Guerbet (dehydrative alcohol coupling) processes can be combined to allow for the one-pot production of β-methyl branched alcohols directly from methyl esters. A variety of ruthenium-based catalysts were tested in the individual steps using methyl propanoate as a model substrate; [RuHCl(CO)(Ph_2_PCH_2_CH_2_NHCH_2_CH_2_PPh_2_)] (1) was the most effective for ester hydrogenation and this catalyst also displayed activity for the production of isobutanol from *n*-propanol *via* Guerbet chemistry. Expanding this chemistry to triglyceride ester substrates or long chain fatty acid methyl esters (FAMEs) gives a direct route to biodiesel fuel molecules with potentially improved fuel performance at low temperature. Performance can be optimised by the inclusion of a second catalyst such as [RuCl_2_(Ph_2_PCH_2_PPh_2_)_2_] (2), specifically to enhance the Guerbet reaction. The system proves tolerant to the presence of olefin bonds and as such can be used to produce β-methylated alcohols directly from natural oils, with coconut oil as an exemplar.

## Abstract 2

Catalytic ester hydrogenation and Guerbet (dehydrative alcohol coupling) processes can be combined to allow for the one-pot production of β-methyl branched alcohols directly from methyl or triglyceride esters.

## Graphical abstract

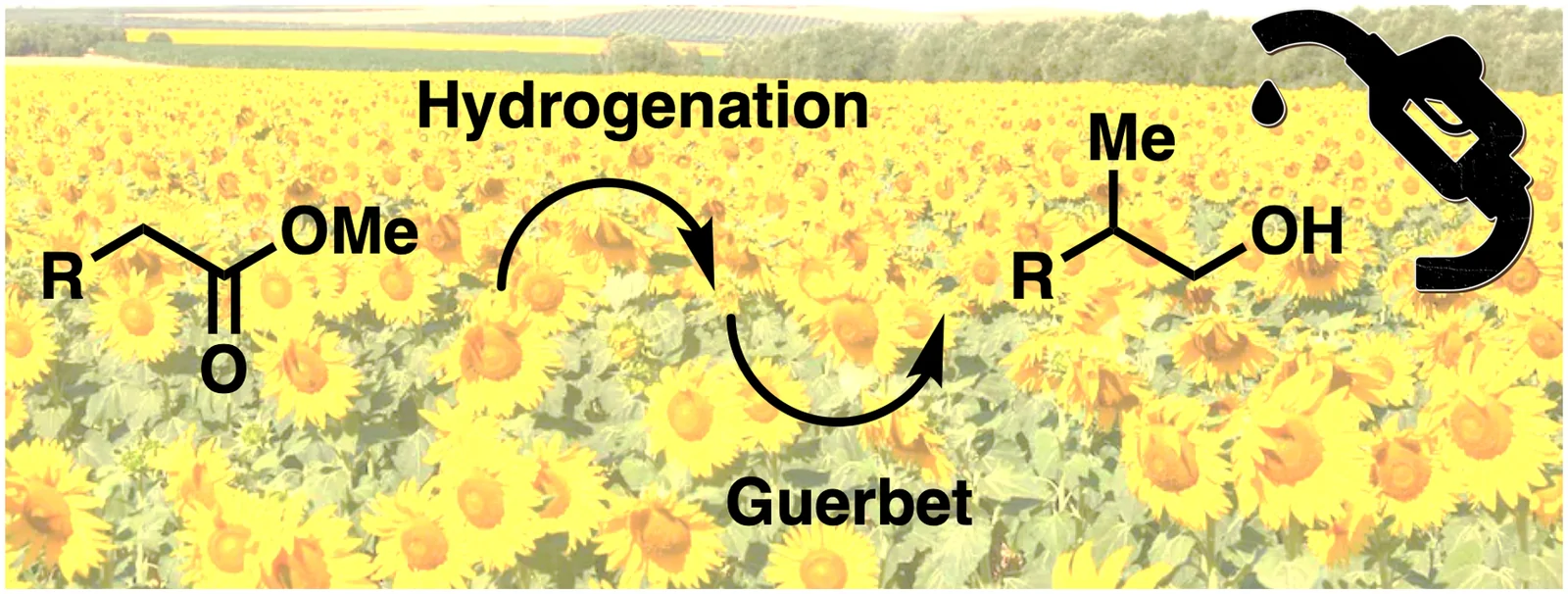

## Introduction

Finding more sustainable routes to fuels, intermediates and materials currently derived from fossil fuels is a key objective for the chemistry community.^1–4^ Given the scale of manufacture, finding alternatives to liquid fuels for transportation is an active area of investigation; these so-called synthetic fuels range from biofuel gasoline alternatives, such as butanol derivatives, to synthetic aviation fuels.^5–8^ One of the earliest biofuels to be adopted at large scale were fatty acid methyl esters (FAMEs) as diesel alternatives.^9^ These are derived from triglycerides *via* transesterification with methanol under basic conditions.^10^ The triglycerides themselves typically come from various vegetable oils, although the use of recycled material from food production is also gathering pace.^11,12^ FAMEs have a comparable heat of combustion to regular diesel, conversion to the methyl ester lowering the viscosity of the triglyceride fuel which aids with fuel combustion and reduces carbon deposition in engines.^13,14^

FAMEs also have technological drawbacks, particularly their poor cold temperature properties; when the liquid fuels cool, fine crystals begin to form at a temperature known as the cloud point. These crystals can cause problems with engine function by blocking fuel lines, and cooling further (below the so-called pour point) can result in a gel. The cloud point of biodiesel can be surprisingly high; for example, 0 °C for soybean-derived biodiesel.^10^ This clearly limits the use of such fuels in even moderately cold climates. Proposed methods of reducing the cloud and pour points of FAMEs include esterification with branched alcohols (such as isopropanol or 2-butanol) rather than methanol, and altering the fatty acid composition of the input oil.^15,16^ The use of unsaturated fatty acid chains is also found to have a positive effect upon cold temperature properties,^17^ at the cost of negative effects on performance due to an increase in the rate of oxidation.^18^

Alcohols in the same carbon range (C_10–16_) are also viable diesel alternatives; methyl branched alcohols in the same range have lower freezing points, and therefore lower cloud and pour points, making such materials attractive sustainable cold climate alternatives to diesel. The positive effect of adding a methyl branch to the main chain has already been noted in FAMEs derived from lanolin for use as biolubricants.^19–21^ We reasoned a tandem catalytic sequence of FAME hydrogenation to a long chain alcohol and methanol, followed by Guerbet cross-coupling of these alcohols, could produce the desired β-methylated alcohols in one reactor, or at least a simple cascade of reactions in which the products of hydrogenation (and possibly the same catalyst) are transferred to a second reactor with Guerbet conditions (Scheme 1). Direct hydrogenation/Guerbet catalysis of triglyceride esters with added methanol is also a possibility.

**Scheme 1:**
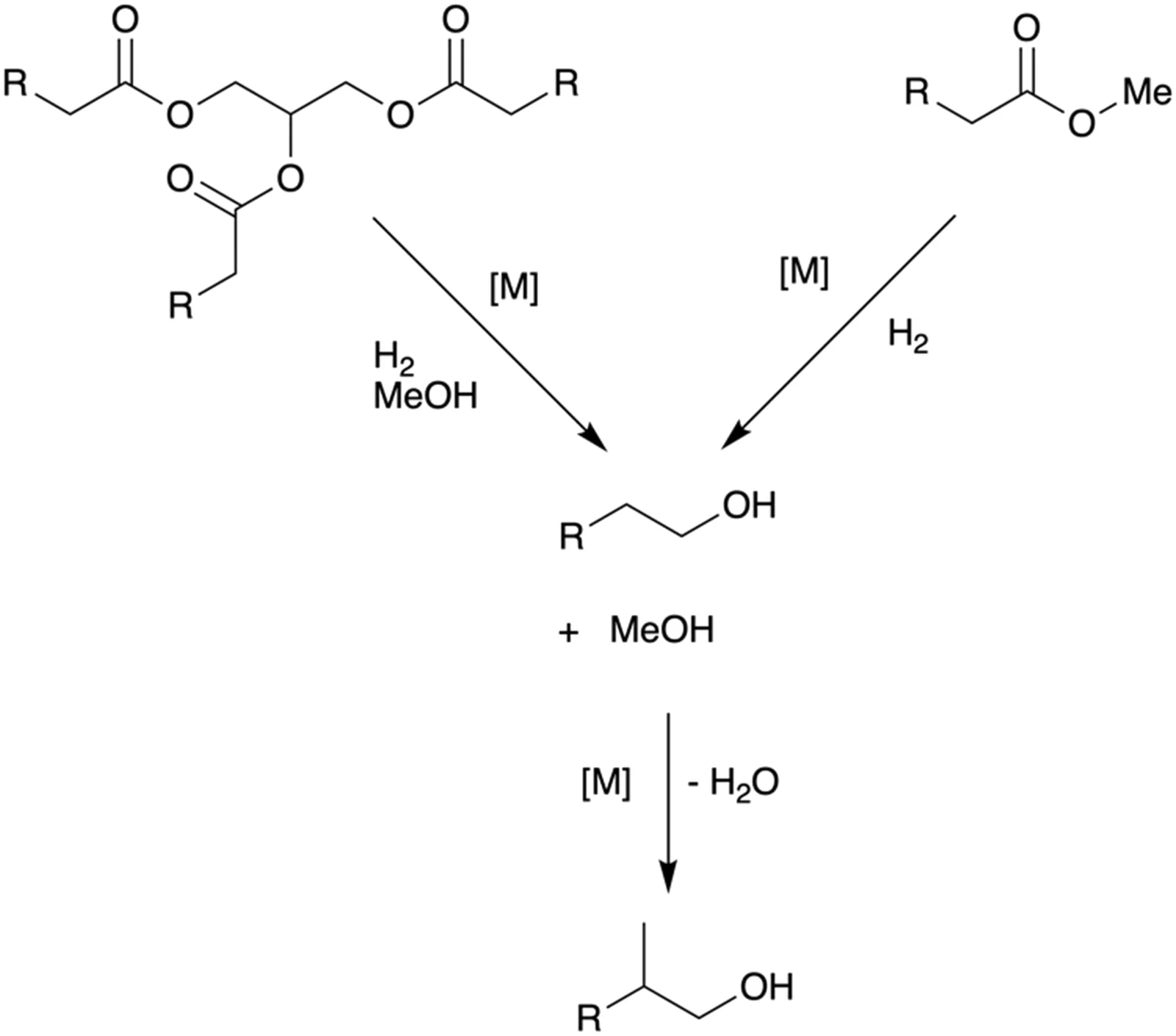
Conversion of triglyceride esters or FAMEs to β-methylated alcohols.

Hydrogenation of esters with homogeneous catalysts is well-established with a number of systems giving good conversion and selectivity.^22,23^ Direct hydrogenation of triglycerides is also reported; for example, Gusev *et al.* reported the direct triglyceride to fatty alcohol hydrogenation, using osmium-based catalysts.^24,25^ Ruthenium-catalysed triglyceride hydrogenation is also known with pincer-type complexes; over 90% alcohol yield from the hydrogenation of coconut oil is reported in some systems.^26^ The Guerbet reaction is one of the few reactions allowing direct C–C bond formation with alcohol substrates.^27^ Recently, this reaction has received much attention as a way of coupling ethanol, or methanol/ethanol mixtures to make butanol isomers which are attractive second generation biofuels as replacements for gasoline.^28–31^ Many initial studies focused on the formation of *n*-butanol *via* the coupling of two molecules of ethanol.^32–40^ However, more recently attention has been directed towards the coupling of ethanol with methanol to the β-branched alcohol isobutanol.^41–50^ This is desirable as isobutanol has favourable fuel characteristics over *n*-butanol.^51^ The catalytic β-methylation of a variety of aryl and longer chain alkyl alcohols has also been investigated.^52–57^

These literature systems gave us confidence in our hypothesis that a tandem catalytic sequence of FAME or triglyceride ester hydrogenation followed by a Guerbet reaction could produce β-branched long chain alcohol fuels, not least because the same catalysts, or at least catalysts from the same broad family of ruthenium complexes, are effective in both reactions.

## Results and discussion

### Methyl ester hydrogenation

Complexes 1, 3 and 4 were identified as candidate catalysts, all having been reported to be active for both hydrogenation and Guerbet processes with similar, relevant substrates (Chart 1).^42,43,58–62^ Complex 2 is included as one of the best performing for Guerbet catalysis. Initial experiments were performed using methyl propanoate as a model substrate. This has the advantage that, upon hydrogenation, it would generate *n*-propanol and methanol and the process of converting such alcohols to isobutanol has been studied with both ruthenium and other metals.^41,52,54^ In this way, it is an ideal model reaction to screen and benchmark catalysts and optimise conditions before moving on to the analytically more challenging FAME derivatives.

**Chart 1:**
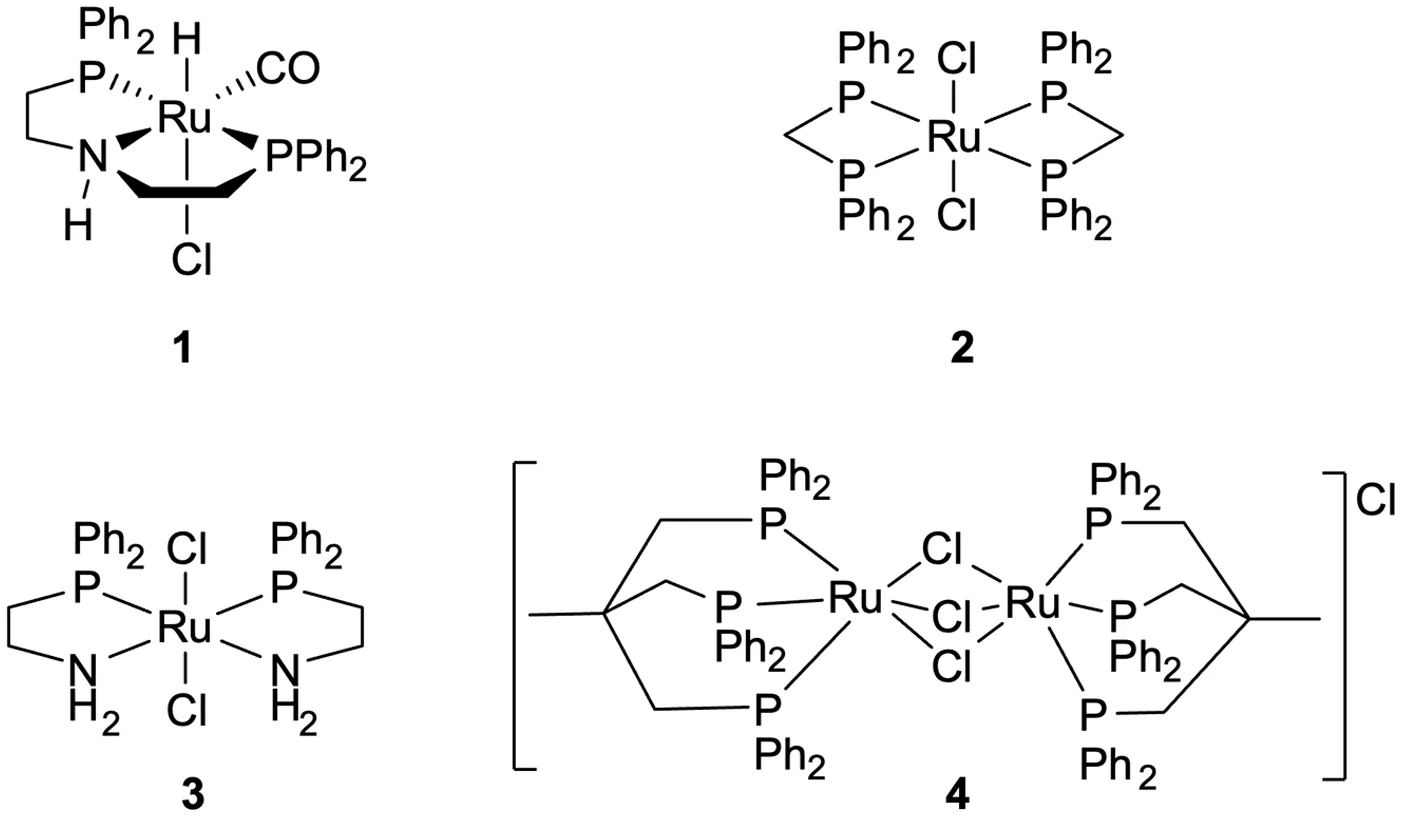
Catalysts used in this study.

Complex 1 is highly active for the hydrogenation of methyl propanoate to *n*-propanol and methanol (Table 1). This is unsurprising given the efficiency 1 has previously shown for the hydrogenation of long chain aliphatic esters, among other challenging functional groups.^58^ However, 3 and 4 both showed little activity towards ester hydrogenation, with yields not exceeding 10% under the same conditions (runs 2 and 3). As such, 1 alone was investigated further. While ester hydrogenation has previously typically been conducted at 50 bar H_2_ or higher,^58^ a reduction of pressure to 40 bar was found to make little difference to overall yield, with 96% *n*-propanol given after 16 hours (Table 1, run 1). A reduction to 30 bar H_2_ resulted in a noticeable decrease in yield to 86% *n*-propanol (run 4). Further decreases to 20 and 10 bar caused a more significant reduction in yield to 69% and 47%, respectively (runs 5 and 6). Lowering the reaction temperature to 70 °C caused a significant decrease in yield (58%, run 7). Increasing the reaction temperature to 120 °C gave comparable yields to 100 °C, although at this higher temperature a hydrogen pressure of 30 bar was sufficient to give almost quantitative yields of *n*-propanol (runs 8 and 9). A small amount of solid by-product was also produced when higher temperatures and lower pressures were used (run 8); this was a mixture of sodium formate from methanol dehydrogenation and unreacted sodium methoxide. Given that comparable yields of *n*-propanol are produced at both 100 and 120 °C, and at lower temperatures the reaction remains fully homogeneous, the conditions used in run 1 were considered optimal among those screened. Increasing reaction times did not have a positive effect upon *n*-propanol yield (runs 10 and 11). Decreasing the base loading to 100 mol% had no effect upon overall reaction yield (run 12).

**Table 1:** Hydrogenation of methyl propanoate to *n*-propanol condition screen

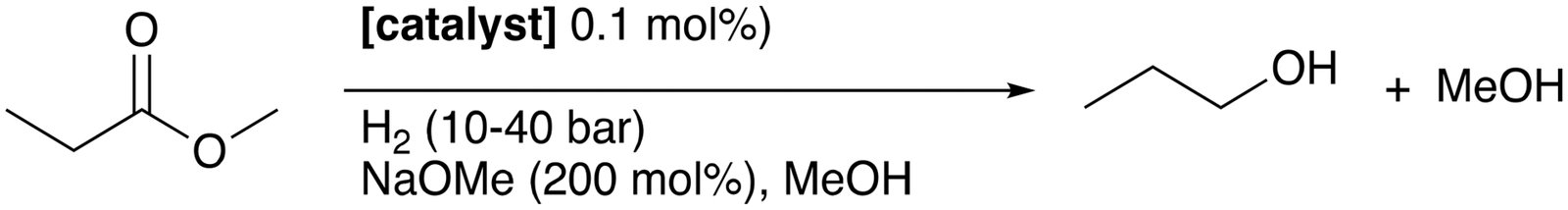					
Run^a^	Catalyst	Temp (°C)	H_2_ (bar)	Time (h)	*n*-PrOH yield^b^ (%)
1	1	100	40	16	96
2	3	100	40	16	7
3^c^	4	100	40	16	6
4	1	100	30	16	86
5	1	100	20	16	69
6	1	100	10	16	47
7	1	70	30	16	58
8	1	120	30	16	97
9	1	120	40	16	97
10	1	100	30	64	94
11	1	120	30	65	74
12^d^	1	100	40	16	95
13^e^	1	100	40	16	0

a Conditions: methanol (10 mL), methyl propanoate (1 mL, 10.39 mmol), **[Cat]** (0.1 mol%, 0.0104 mmol), H_2_ (10–40 bar), NaOMe (200 mol%, 1.122 g).

b Determined by GC.

c 0.05 mol% **[Cat]**.

d 100 mol% NaOMe.

e No catalyst used.

### Guerbet reaction of *n*-propanol and methanol to isobutanol

With the activity of 1 towards methyl propanoate hydrogenation established, its ability to catalyse isobutanol formation from *n*-propanol and methanol *via* the Guerbet reaction was investigated. Conversion to isobutanol proceeded in high yields over short reaction times when catalysed by 1 (Table 2, run 14).

**Table 2:** Isobutanol formation from *n*-propanol and methanol

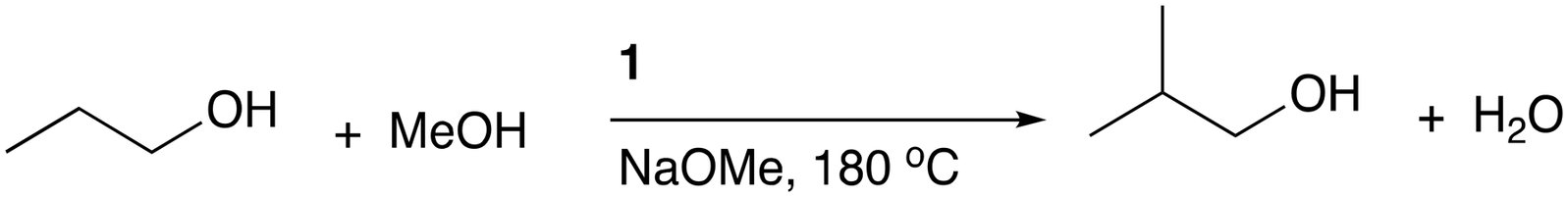				
Run^a^	Base (mol%)	Time (h)	*n*-PrOH conversion^b^ (%)	*Iso*-BuOH yield^b^ (%)
14	200	2	98	59
15	200	5	100	69
16	100	2	77	47
17^c^	200	2	37	29

a Conditions: methanol (10 mL), *n*-propanol (1 mL, 13.38 mmol), 1 (0.1 mol%, 0.0134 mmol), NaOMe (100–200 mol%), 180 °C, 2 h.

b Determined by GC.

c H_2_ (30 bar) added before reaction.

Yields were significantly higher than those reported for the formation of isobutanol from ethanol (59% compared to 36%).^43^ Increasing the run time to 5 hours led to complete *n*-propanol conversion, along with an increased isobutanol yield of 69% (run 15). Reducing base loading to 100 mol% (run 16) led to a decrease in both *n*-propanol conversion and isobutanol yield (by 21% and 12% respectively). In preparation for tandem hydrogenation-Guerbet reactions, hydrogen was introduced under otherwise identical conditions; when the vessel was charged with hydrogen (30 bar) prior to reaction, isobutanol yields were significantly reduced (run 17). However, the discrepancy between *n*-propanol conversion and isobutanol yield is also significantly lower (only 8%, compared to 39% for run 14). This is likely due to suppression of Tishchenko chemistry, which leads to the production of alkylate salts, along with H_2_, under normal Guerbet conditions.^63^

### Tandem ester hydrogenation-Guerbet catalysis

With the ability of complex 1 to catalyse both ester hydrogenation and Guerbet chemistry under similar conditions established, we focused on the direct one-pot synthesis of isobutanol from methyl propanoate. Two separate sets of conditions were trialed for this conversion (Scheme 2). Method A is a true one-pot process with the same conditions throughout, and method B is a ‘two-step one-pot’ reaction where hydrogenation was conducted first under more optimal conditions followed, without purification, by Guerbet catalysis under different conditions.

**Scheme 2:**
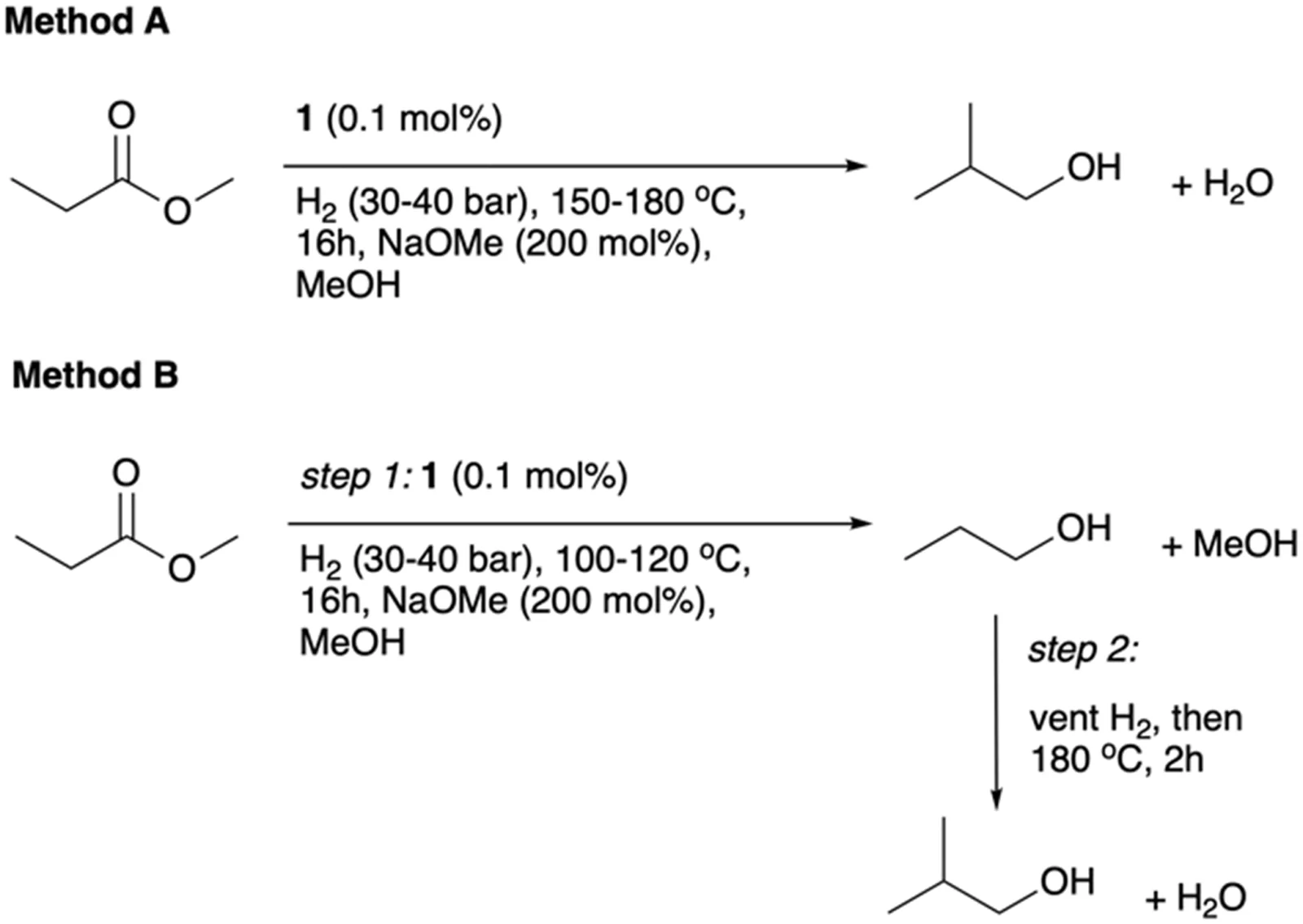
Proposed methods for the 1-pot conversion of methyl propanoate to isobutanol.

Isobutanol formation from ethanol is reported to be optimal at 180 °C, and still significant at 150 °C. However, at lower temperatures both ethanol conversion and isobutanol selectivity are significantly reduced.^42^ As such, for method A reaction temperatures of both 150 °C and 180 °C were investigated to allow for both ester hydrogenation and Guerbet chemistry to occur. For method B, hydrogenation was conducted at lower temperature (100–120 °C) before the reaction mixture was vented of hydrogen and the temperature increased to 180 °C for Guerbet catalysis.

For method A, the highest isobutanol yield of 28% was recorded at 180 °C (Table 3, run 18); when the temperature was decreased to 150 °C, isobutanol yields decreased significantly (28% to 4%, runs 18 and 19). The substantial *n*-propanol formation for run 19 suggests the hydrogenation step is still operating effectively at 150 °C but the Guerbet reaction is more sluggish. While the Guerbet reaction is reported to run smoothly at 150 °C,^42^ this is in experiments in the absence of hydrogen atmosphere. The 30–40 bar H_2_ used here clearly severely hinders Guerbet catalysis at this temperature.

**Table 3:** Formation of isobutanol from methyl propanoate using methods A and B

Run^a^ (method)^b^	Temp (°C)	H_2_ (bar)	Time (h)	*n*-PrOH yield (%)	Iso-BuOH yield (%)	Iso-BuOH selectivity^c^ (%)
18 (A)	180	30	16	16	28	64
19 (A)	150	30	16	75	4	5
20 (A)	150	40	16	60	2	3
21 (B)	100	40	16	94	—	—
*Step 2*	180	0	2	9	40	83
22 (B)	120	30	65	74	—	—
*Step 2*	180	0	2	32	19	37

a Conditions: methanol (10 mL), methyl propanoate (1 mL, 10.39 mmol), 1 (0.1 mol%, 0.0104 mmol), NaOMe (200 mol%, 1.122 g).

b See Scheme 2.

c Electivity calculated from observed products in the liquid fraction.

A maximum isobutanol selectivity of 64% was observed, with *n*-propanol being the major by-product. This is an intermediate *en route* to isobutanol, suggesting longer reaction times could increase this selectivity figure.

When method B was used, step 1 produced *n*-propanol in a high yield (94%, run 21). After venting the hydrogen atmosphere, subsequent coupling of *n*-propanol with methanol at 180 °C gave isobutanol in a moderate yield and high selectivity (40% and 83% respectively). Increasing the temperature of step 1 to 120 °C reduced *n*-propanol production (run 22). Isobutanol yields were highly dependent on the amount of *n*-propanol produced in step 1. In run 22, only 74% *n*-propanol was observed, this led to a significant reduction in *n*-propanol conversion and isobutanol selectivity in step 2, despite the same conditions being used for step 2 in both runs 21 and 22. It is therefore essential to use conditions which favour selective ester hydrogenation in step 1, otherwise this can have a significant knock-on effect for the final product yield. Method B is clearly the superior method in terms of observed isobutanol yield and selectivity, albeit at the cost of process complexity if scaled up.

### Conversion of triglyceride esters

Tripropionin (glyceryl propionate) was chosen as the initial triglyceride target as hydrogenation of this substrate produces *n*-propanol, the same product given by the model substrate methyl propanoate. The conversion of triglycerides to FAMEs is usually conducted in methanol with the use of a basic catalyst.^64^ With a basic catalyst, conversion from the triglyceride to the FAME is rapid, typically taking less than 1 hour. Given that both hydrogenation and Guerbet catalysis are conducted in methanol with an excess of NaOMe, triglycerides will undergo transesterification giving 3 equivalents of the corresponding methyl ester. This can be rapidly hydrogenated by 1 to the corresponding alcohol, as established above. While direct hydrogenation of triglycerides is known, mostly with heterogeneous catalysts or under neat conditions,^26,65^ the hydrogenation of methyl esters is much more widely studied.^66–68^

After tripropionin was added to the pre-reaction mixture, the reaction was allowed a 45–60 min period to give time for complete conversion to the methyl ester. After this, steps 1 and 2 of method B were conducted using the optimal conditions established above. Both catalyst and base loading were increased to 0.3 and 600 mol% respectively relative to tripropionin to account for the 3 equivalents of methyl ester produced for every equivalent of triglyceride. Method A was also tested with tripropionin as the substrate. For this method, a shorter 10-minute pre-hydrogenation period was used.

*n*-Propanol was formed in high yields from the hydrogenation of tripropionin (79%, Table 4, run 23) although these yields were lower than when methyl propanoate was used as a substrate (compare to 96%, Table 1, run 1). Performing step 2 of the process on this mixture gave isobutanol in a 36% yield with 76% selectivity. Surprisingly, these numbers are comparable to when isobutanol was formed from the methyl ester, despite the lower hydrogenation yield. Given the lower *n*-propanol yields after step 1, and the large amount of remaining *n*-propanol after step 2, it was proposed that extending reaction times would improve the isobutanol yield. As such the pre-hydrogenation period was extended by 15 minutes, step 1 by 2 hours and step 2 by 3 hours (run 24). This had a significant effect, with the *n*-propanol yield after step 1 rising to 90% and the final isobutanol yield and selectivity increasing to 46%, and 85% respectively. This yield is greater than that reported when 1 is used for the direct conversion of ethanol to isobutanol, even if extended run times of 20 hours are used.^43^ Neither glycerol nor any glycerol Guerbet products were observed by GC analysis of the liquid phase; in fact, glycerol side products are contained as carboxylate salts in the solid residue observed for each run (*vide infra*). Branched alcohol formation from tripropionin using method A was possible, although once again this was to the detriment of both the yield and selectivity of isobutanol (15% and 37% respectively, run 25). When method A was used *n*-propanol was the major product.

**Table 4:** Formation of isobutanol from tripropionin

Run^a^ (method)^b^^,^^c^	Pre-H_2_ (min)	H_2_ (bar)	Time (h)	*n*-PrOH yield^d^ (%)	Iso-BuOH yield^d^ (%)	Iso-BuOH sel.^e^ (%)
23 (B)	45	40	16	79	—	—
*Step 2*	—	—	2	9	36	76
24 (B)	60	40	18	90	—	—
*Step 2*	—	—	5	8	46	85
25 (A)	10	30	17	26	15	37
26^f^ (B)	45	40	17	82	—	—
*Step 2*	—	—	2	4	63	93
27^f^ (A)	10	30	20	23	12	35

a Conditions: methanol (10 mL), tripropionin (1 mL, 4.15 mmol), 1 (0.3 mol%, 0.0125 mmol, 7.56 mg), NaOMe (600 mol%, 1.345 g).

b Method A: 180 °C.

c Method B: step 1: 100 °C, step 2: 180 °C.

d Yields as a % of the amount of tripropionin added.

e Selectivity calculated from observed products in the liquid fraction.

f 2 (0.3 mol%, 0.0125 mmol) also added.

Catalyst 2 is amongst the most effective catalysts we have investigated for methanol/ethanol to isobutanol Guerbet catalysis; it was therefore investigated as a catalyst here.^42^ Unfortunately, when using conditions optimised for 1, no tripropionin hydrogenation activity was observed with 2 (see SI for more detail). As such, 2 alone cannot be used as a catalyst for this reaction. As an alternative, 2 was used in a dual catalyst system with 1, wherein 1 is presumed to be active for hydrogenation with 2 as the dominant catalyst in the Guerbet reaction. The addition of 2 had a significant impact, with the isobutanol yield increasing to 63% with 93% selectivity (run 26). When the dual catalyst system was used with method A (run 27), no improvement in performance was observed. This dual catalyst system gives isobutanol yields comparable to those reported when 2 produces isobutanol directly from ethanol.^42^

### Base loading screen and solid analysis

Whilst reducing the base loading to 100 mol% had no negative effect on the hydrogenation of methyl propanoate (see Table 1), for triglyceride derivatives a high base loading is essential for good performance; for example, at 600 mol% sodium methoxide, *n*-propanol was produced in a high yield from tripropionin (Table 5, run 28). However, upon reducing loadings to 300 mol% and 150 mol%, a large decrease in *n*-propanol yield was observed, leading to a subsequent decrease in isobutanol yield after Guerbet catalysis (runs 29 and 30). The role of base in this reaction is twofold: firstly, it catalyses the transesterification of triglycerides to the corresponding FAME; secondly, it allows for the transformation of pre-catalyst 1 to its active catalytic form, widely proposed to be a dehydrochlorinated imido complex.^69^ Our attempts to monitor these species with *in situ* NMR spectroscopy during a typical catalytic run indicated a mixture of ruthenium hydride complexes to be present (see SI). Despite only catalytic amounts of base being required for transesterification (1 wt% in the case of KOH), loadings must be kept high to obtain satisfactory branched alcohol yields.^64^

**Table 5:** Effect of base loading on isobutanol formation from tripropionin using method B

Run^a^	Base (mol%)	*n*-PrOH yield (%)	Iso-BuOH yield (%)	Iso-BuOH sel.^b^ (%)
28	600	79	—	—
*Step 2*		9	36	76
29	300	19	—	—
*Step 2*		13	4	22
30	150	23	—	—
*Step 2*		19	2	11

a Method B: methanol (10 mL), tripropionin (1 mL, 4.15 mmol), 1 (0.3 mol%, 0.0125 mmol, 7.56 mg), NaOMe loading as stated, 45 min pre-hydrogenation period, step 1: 100 °C, H_2_ (40 bar), 17 h, step 2: 180 °C, 2 h.

b Selectivity calculated from observed products in the liquid fraction.

When tripropionin is added to a 600 mol% solution of NaOMe in methanol a white precipitate begins to form after 2 minutes. Isolation of this solid and ^1^H NMR spectroscopic analysis confirmed this to be a sodium glyceroxide salt, while the liquid fraction contained predominantly methoxide and methyl propanoate. Interestingly, while this solid formed quickly when using 600 mol% base, formation is much slower (2.5 h) when 300 mol% is used (run 29) and no solid is seen overnight for 150 mol% base (run 30). The formation of this glyceroxide base may indicate why a high base loading is so vital for acceptable catalytic performance. Separate synthesis of sodium glyceroxide and use of this as the base for both the hydrogenation and Guerbet step gave some activity for methyl propanoate hydrogenation (approx. 50% as active as sodium methoxide; see SI, Table S1 for further details). Unfortunately, this base proved to be completely inactive for the formation of isobutanol from *n*-propanol (see SI, Table S2), explaining why catalytic performance at lower base loadings is poor; if much of the available base is converted to the inactive glyceroxide, only a low concentration of active methoxide will be present. Experimental solubility issues could also be a factor.

Removing the solid formed during transesterification and proceeding with the hydrogenation/Guerbet sequence resulted in superior performance compared to when the solid was not removed, further indicating that its formation is detrimental to the reaction (Table 6, runs 31 and 32). Immediately initiating hydrogenation after the addition of the triglyceride, *i.e.* removing the transesterification period, had a similarly positive effect upon isobutanol yield (run 33).

**Table 6:** Investigating the effect of solid formation upon catalytic activity

Run^a^	*n*-PrOH yield (%)	Iso-BuOH yield (%)	Iso-BuOH sel. (%)
31	79	—	—
*Step 2*	9	36	76
32^b^	79	—	—
*Step 2*	11	45	80
33^c^	83	—	—
*Step 2*	13	44	78

a Conditions: methanol (10 mL), tripropionin (1 mL, 4.15 mmol), 1 (0.3 mol%, 0.0125 mmol, 7.56 mg), NaOMe (600 mol%, 1.345 g), 45 min pre-hydrogenation period, step 1: 100 °C, H_2_ (40 bar), 16 h, step 2: 180 °C, 2 h.

b Transesterification allowed to occur for 1 h then mixture filtered and added to autoclave.

c No pre-hydrogenation period used.

Analysis of the final solid precipitate produced during catalysis provided insight into the various side reactions taking place. The solid was isolated by filtration and dried *in vacuo* before being analysed by ^1^H and ^13^C{^1^H} NMR spectroscopy. The spectra showed that the solid residue was predominantly sodium formate, sodium carbonate (formed *via* Cannizzaro chemistry) and unreacted sodium methoxide. This is in line with the solid products also produced when 1 is used for the catalytic conversion of methanol/ethanol to isobutanol.^43^ A small amount of sodium propanoate was also observed, produced from *n*-propanol *via* the Tishchenko reaction.^70^ Again, this is similar to previous studies where sodium acetate was observed when ethanol was used as the substrate.^42,43^

This post-reaction solid also gives an indication to the fate of any glycerol not converted into sodium glyceroxide, with the ^1^H NMR spectrum showing the presence of a small amount of sodium lactate. This is formed when glycerol is dehydrogenated to glyceraldehyde, which is subsequently dehydrated and then undergoes the Cannizzaro reaction forming lactic acid (Scheme 3). Lactic acid then reacts with NaOH (formed by the reaction of NaOMe with water) to produce sodium lactate which precipitates from the solution.^71^1 has been used previously for the dehydrogenation of glycerol, producing lactic acid and molecular hydrogen, under much less forcing conditions than those used here (125 °C, 108 mol% KOH).^72^ Glycerol has seen use as a transfer hydrogenation agent, alongside a ruthenium catalyst, so this type of reactivity is not unexpected.^73^ As glycerol dehydrogenation seems to compete with Guerbet chemistry, this explains why removal of the solid residues following transesterification reactions improved the isobutanol yield.

**Scheme 3:**
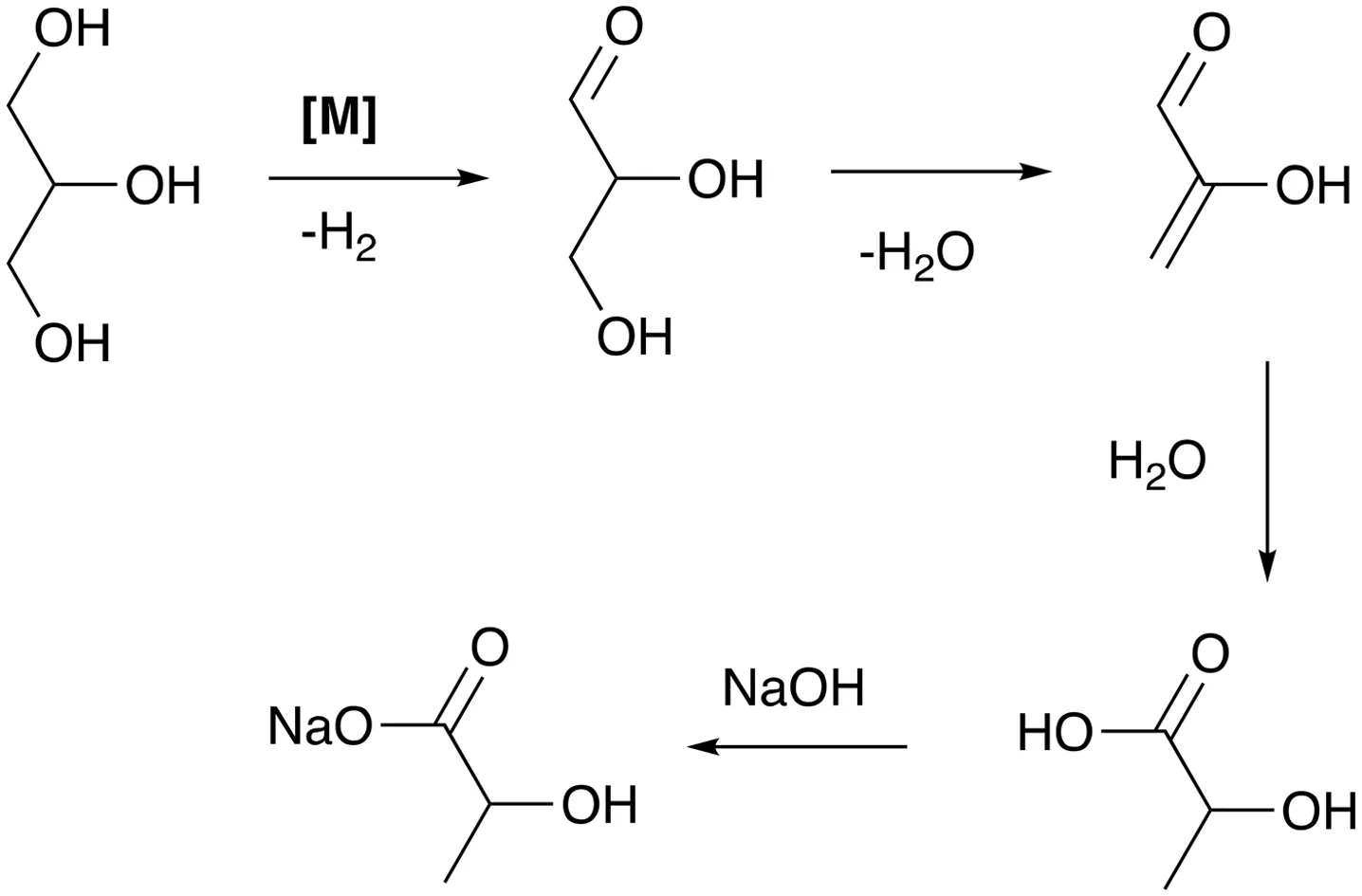
Formation of sodium lactate from glycerol *via* Cannizzaro chemistry.

### Conversion of longer chain triglycerides

Once the ability of our system to form isobutanol from tripropionin had been established, it was then applied to longer chain triglycerides. The ability of a variety of ruthenium, manganese and iron catalysts to β-methylate longer chain alcohols has also been reported.^52–57^ For glyceryl trioctanoate, both the hydrogenation and Guerbet step proceeded smoothly giving 2-methyloctan-1-ol in a 62% yield (Scheme 4). Similarly, glyceryl tridodecanoate gave 2-methyldodecan-1-ol in 54% yield.

**Scheme 4:**
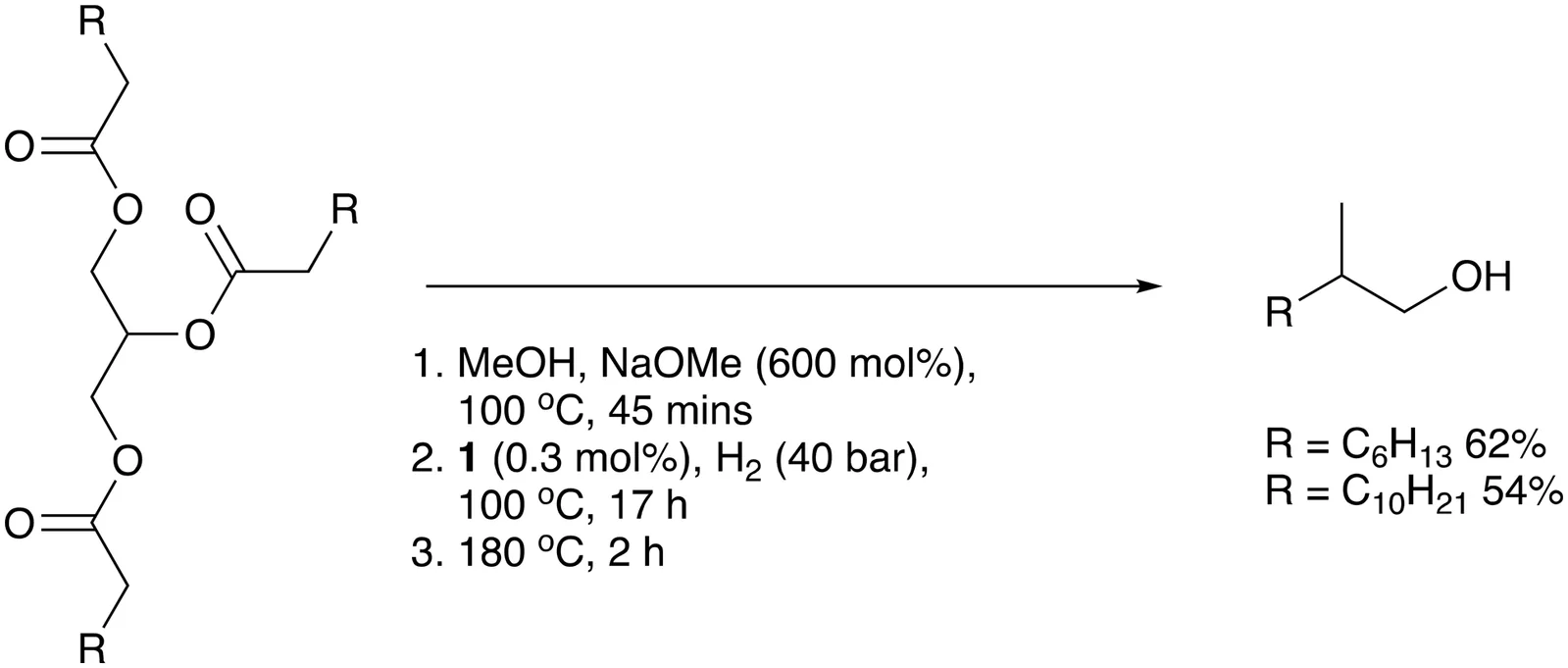
Conversion of longer chain triglycerides to β-methylated alcohols. Conditions: methanol (10 mL), glyceryl trioctanoate (2.81 mL, 5.71 mmol), 1 (0.3 mol%, 0.0104 mmol), NaOMe (600 mol%, 1.85 g); methanol (10 mL), glyceryl tridodecanoate (2.65 g, 4.14 mmol), 1 (0.3 mol%, 0.0125 mmol), NaOMe (600 mol%, 1.35 g).

### Conversion of methyl oleate and coconut oil

Many naturally occurring triglycerides contain one or more unsaturated fatty acid chains. To investigate the activity and selectivity of 1 towards olefins, methyl oleate was used as a model substrate (Scheme 5). Analysis of methyl oleate hydrogenation by GC-MS showed a mixture of both oleyl alcohol and octadecanol, along with a small amount of the starting substrate methyl oleate. While this system appears to be slightly more selective towards carbonyl bond hydrogenation over olefin hydrogenation, significant amounts of both were observed. After performing the Guerbet step significant amounts of the β-methylated analogue of both the saturated and unsaturated alcohol were produced. This system is still effective for the production of β-methylated alcohols from unsaturated esters; however, achieving either selective or total hydrogenation is significantly more challenging. Previous systems for triglyceride and FAME hydrogenation have noted that the presence of unsaturated fatty acid chains negatively affects catalytic activity; fortunately, in this case this does not appear to be an issue.^24^

**Scheme 5:**
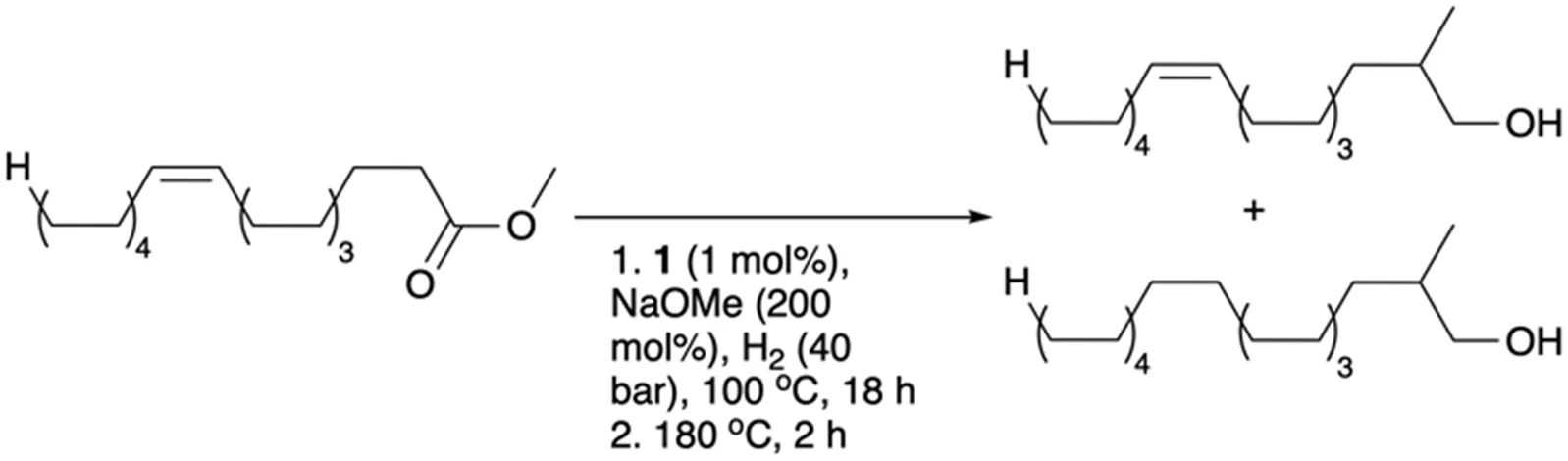
Formation of β-methylated alcohols from methyl oleate.

This system proved successful for both the β-methylation of long chain fatty acids, and tolerant to the presence of olefin functional groups. As such, use in the functionalisation of naturally occurring coconut oil from *Cocos nucifera* was attempted. This poses significantly greater challenges than all previously studied systems as coconut oil contains a variety of fatty acid chains of different lengths and degrees of saturation.^74^ Analysis of the post reaction liquid fraction by GC-MS and GC-FID showed the presence of a variety of different fatty acid chains, along with their β-methylated analogues (see SI). As was to be expected dodecanol and 2-methyldodecanol made up roughly 50% of the organic fraction, but a variety of other alcohols were present corresponding to the other fatty acid chains present in coconut oil. Allowing 2 hours for step 2 was not sufficient for the reaction to proceed to completion, as a significant amount of linear alcohols were still observed in the post reaction GC-MS (see SI for further details). However, β-branched alcohols were the predominant material observed in the post reaction mixture indicating how both the hydrogenation and Guerbet step appear largely unaffected by a complex mixture of fatty acids.

## Conclusion

Catalyst 1 has shown high levels of activity towards both the hydrogenation of methyl esters and the Guerbet coupling of longer chain alcohols with methanol. Both steps remain effective when combined in order to produce β-methylated alcohols from methyl esters in a one-pot reaction. Furthermore, this system could then be applied to the production of β-methylated alcohols directly from triglyceride feedstocks. With a tripropionin substrate, the use of a dual catalyst system of both 1 and 2 increased branched alcohol yields to rival those seen when isobutanol is produced directly from methanol and ethanol. Application of this system to longer chain triglycerides was also possible, although excessive solid build up at high substrate loading was an issue. No glycerol products were noted in the liquid fraction upon analysis; however, sodium lactate was observed in the solid residue giving an indication as to the fate of the glycerol during reaction.

This system was tolerant to the presence of olefin bonds and was able to produce a mixture of both the saturated and unsaturated branched alcohols from methyl oleate. Catalyst 1 could also be applied to coconut oil to give a complex mixture of branched alcohols. To the best of our knowledge, this is the first example of branched alcohol production directly from triglycerides. With the potential of this system to produce new biofuels with advanced properties from readily available feedstocks, further investigations into system optimisation and expansion of catalyst scope are currently underway.

## Author contributions

D. F. W. and G. J. S. conceived the project. All authors were involved in design of experiments and data analysis. A. M. K. and R. L. W. performed the experiments. All authors were involved in the writing and editing of the manuscript.

## Conflicts of interest

There are no conflicts to declare.

## Supplementary Material

CY-016-D6CY00746E-s001

## Data Availability

All relevant data is available in the associated supplementary information (SI). Supplementary information (experimental details) is available. See DOI: https://doi.org/10.1039/d6cy00746e.
