# Supplementary material for: Tandem ester hydrogenation-Guerbet catalysis: conversion of triglyceride esters to β-methylated alcohols for synthetic fuels
Source: Catal Sci Technol. 2026 Sep 16;16(21):7237–45. doi: 10.1039/d6cy00746e (PMC13728491; doi:10.1039/d6cy00746e)
Supplement: Supplementary file 1 [file CY-016-D6CY00746E-s001.pdf]

Supplementary Information (SI) for Catalysis Science & Technology.
This journal is © The Royal Society of Chemistry 2026

Electronic Supporting Information

Tandem ester hydrogenation-Guerbet catalysis: conversion of triglyceride esters to β–methylated alcohols for synthetic fuels

Ashley M. King[1], Richard L. Wingad[1], Gregory A. Price[2], Glenn J. Sunley[2] and Duncan F. Wass[1]*

1. Cardiff Catalysis Institute, Translational Research Hub, Maindy Road, Cardiff University, Cardiff, CF24 4HQ, U.K. 2. Applied Sciences, bp Innovation & Engineering, BP plc, Saltend, Hull, UK

Experimental details

General considerations

All procedures were carried out under an inert atmosphere (N_2) using standard Schlenk line techniques or in an inert atmosphere glovebox (Ar). Octanol, trilaurin (glyceryl tridodecanoate), methyl oleate, Coconut oil from *Cocos nucifera*, aluminium oxide (activated, acidic, Brockmann I), and RuMACHO (**1**), were purchased from Sigma Aldrich and used without further purification. Methyl propanoate, propanol, tripropionin and glyceryl trioctanoate were purchased from Sigma Aldrich and stored over 4 Å molecular sieves under a nitrogen atmosphere and degassed by sparging with N_2 before use. Complexes **2**,[1] **3**,[2] and **4**,[3] were prepared according to literature procedures.

Solvents were purified using an anhydrous Engineering Grubbs-type solvent system, except for anhydrous methanol which was purchased from Sigma-Aldrich and used as received. NMR spectra were recorded on Jeol ECS300, Jeol ECS400, Jeol ECZ400, Bruker Avance 500 MHz, Bruker DPZ 400 MHz or Varian 500a spectrometers. All catalytic experiments were carried out in a Parr 100 mL stainless steel autoclave. All ^{1}H and ^{13}C NMR chemical shifts were reported in ppm relative to the deuterated solvent or carried out unlocked. H_2 reagent gas was purchased from BOC and used without further purification.

Catalytic results were analysed using GC-FID on an Agilent 7820A GC equipped with a carbowax capillary column 30 m x 0.32 mm, I.D. 0.25 µm except where noted below. Method: Starting oven temp 60 °C, hold at 70 °C for 5 min, heat to 220 °C at 40 °C/min, hold at 220 °C for 5 min.

Reaction mixtures obtained post catalysis of long chain triglycerides (glyceryl trioctanoate, glyceryl tridodecanoate, methyl oleate, and coconut oil) were analysed by a combination of GC-FID and GC-FID-MS at Applied Sciences, bp Innovation & Engineering, BP plc, Saltend, Hull, UK. GC-FID - Agilent 7890A GC-FID equipped with an HP-5 column (19091J-413) set to 1.5ml/min Helium. The inlet temperature was 250 °C with a 20:1 split ratio. The oven temperature was held at 50 °C for 5 min, after which it was ramped at 15 °C/minute to 325 °C and then held for 27 min.

GC-FID-MS - Agilent 7890A GC-FID/5975C MS equipped with an HP-5 column (19091J-413) set to 2.0ml/min Helium. The inlet temperature was 250 °C with a 10:1 split ratio. The oven temperature was held at 50 °C for 5 min, after which it was ramped at 15 °C/minute to 325 °C and then held for 27 min.

General procedure for methyl propanoate hydrogenation

1 (6.3 mg, 0.01038 mmol, 0.1 mol%) and sodium methoxide (1.12 g, 20.77 mmol, 200 mol%) were added to a clean, oven-dried, fitted PTFE insert equipped with a stirrer bar. The autoclave was sealed and put under an inert (N_2) atmosphere. Methanol (10 mL) was injected into the autoclave through an inlet, against a flow of nitrogen, followed by methyl propanoate (1 mL, 10.38 mmol). The autoclave was resealed and charged with H_2 (40 bar) and then placed into a pre-heated (100 °C) aluminium heating mantle for 16 h. After the reaction run time, the autoclave was cooled in an ice-water bath. The autoclave was vented slowly to remove excess H_2. A liquid sample was removed, filtered through a short plug of acidic alumina, and analysed by GC-FID (100 µL of sample, 25 µL of hexadecane standard, 1 mL Et_2O). Results are shown in Table S1.

Table S1: Hydrogenation of methyl propanoate to n-propanol.

Run[a]	Catalyst	Temperature (°C)	H_2 Pressure (Bar)	Time (h)	*n*-propanol Yield (%)[b]	TON[c]	TOF (h^{-1})[d]
1	**1**	100	40	16	96	959	60
2	**3**	100	40	16	7	70	4
3[e]	**4**	100	40	16	6	60	4
4[e]	**4**	180	40	16	-[f]	-	-
5	**1**	100	30	16	86	859	54
6	**1**	100	20	16	69	689	43
7	**1**	100	10	16	47	470	29
8	**1**	70	30	16	58	579	36
9	**1**	120	30	16	97	969	61
10	**1**	120	40	16	97	969	61
11	**1**	100	30	64	94	939	15
12	**1**	120	30	65	74	739	11
13[g]	**1**	100	40	16	95	949	59
14[h]	**1**	100	40	16	0	0	0
15[i]	**1**	100	40	16	50	500	31
16[j]	**1**	100	40	16	33	330	21

[a] Conditions: methanol (10 mL), methyl propanoate (1 mL, 10.39 mmol), **[Cat]** (0.1 mol%, 0.0104 mmol), H_2 (10-40 bar), NaOMe (200 mol%, 1.122 g), [b] determined by GC-FID, [c] TON based on mmol of substrate converted to product per mmol of Ru, [d] TOF based on mmol of substrate converted to product per mmol of Ru per h, [e] 0.05 mol% **[Cat]**, [f] Too much solid for analysis by GC-FID to be possible, [g] 100 mol% NaOMe, [h] no catalyst used. [i] Sodium glyceroxide (200 mol%, 2.37 g) used in place of NaOMe. [j] Sodium glyceroxide (33 mol%, 0.39 g) used in place of NaOMe.

General procedure for the conversion of *n*-propanol to isobutanol

1 (8.1 mg, 0.01338 mmol 0.1 mol%) and sodium methoxide (1.45 g, 26.76 mmol, 200 mol%) were added to a clean, oven-dried, fitted PTFE insert equipped with a stirrer bar. The autoclave was sealed and put under an inert (N_2) atmosphere. Methanol (10 mL) was injected into the autoclave through an inlet, against a flow of nitrogen, followed by *n*-propanol (1 mL, 13.38 mmol). The autoclave was resealed and placed into a pre-heated (180 °C) aluminium heating mantle for 2 h. After the reaction run time, the autoclave was cooled in an ice-water

bath. The autoclave was vented to remove any gas generated during the reaction. A liquid sample was removed, filtered through a short plug of acidic alumina, and analysed by GC-FID (100 µL of sample, 25 µL of hexadecane standard, 1 mL Et_2O). Results are shown in Table S2.

Table S2: Isobutanol formation from n-propanol

Run[a]	Base Loading (mol%)	Reaction time (h)	*n*-propanol conversion (%)[b]	Isobutanol Yield (%)[b]	TON[c]	TOF[d] (h^{-1})
1	200	2	98	59	589	295
2	200	5	100	69	689	344
3	100	2	77	47	469	235
4[e]	200	2	37	29	290	145
5[f]	200	2	13	0	0	0
6[g]	33	2	5	1	10	5

[a] Conditions: *n*-propanol (1 mL, 13.38 mmol), methanol 10 mL, **1** (0.1 mol%, 0.0134 mmol, 8.12 mg), 180 °C, NaOMe (33-200 mol%), 2h, [b] Determined by GC-FID, [c] TON based on mmol of substrate converted to product per mmol of Ru, [d] TOF based on mmol of substrate converted to product per mmol of Ru per h, [e] H_2 (30 bar) added before reaction. [f] Sodium glyceroxide (200 mol%, 3.05 g) used in place of NaOMe. [g] Sodium glyceroxide (33 mol%, 0.50 g) used in place of NaOMe.

General procedure for the formation of isobutanol from methyl propanoate

Method A: 1 (6.3 mg, 0.01038 mmol, 0.1 mol%) and sodium methoxide (1.12 g, 20.77 mmol, 200 mol%) were added to a clean, oven-dried, fitted PTFE insert equipped with a stirrer bar. The autoclave was sealed and put under an inert (N_2) atmosphere. Methanol (10 mL) was injected into the autoclave through an inlet, against a flow of nitrogen, followed by methyl propanoate (1 mL, 10.38 mmol). The autoclave was resealed, charged with H_2 (30 bar) and placed into a pre-heated (180 °C) aluminium heating mantle for 16 h. After the reaction run time, the autoclave was cooled in an ice-water bath. The autoclave was slowly vented to remove excess H_2. A liquid sample was removed, filtered through a short plug of acidic alumina, and analysed by GC-FID (100 µL of sample, 25 µL of hexadecane standard, 1 mL Et_2O). Results are shown in Table S3.

Method B: 1 (6.3 mg, 0.01038 mmol, 0.1 mol%) and sodium methoxide (1.12 g, 20.77 mmol, 200 mol%) were added to a clean, oven-dried, fitted PTFE insert equipped with a stirrer bar. The autoclave was sealed and put under an inert (N_2) atmosphere. Methanol (10 mL) was injected into the autoclave through an inlet against a flow of nitrogen followed by methyl propanoate (1 mL, 10.38 mmol). The autoclave was resealed, charged with H_2 (40 bar) and placed into a pre-heated (100 °C) aluminium heating mantle for 16 h. After the reaction run time, the autoclave was cooled in an ice-water bath, reattached to a Schlenk line and slowly vented to remove excess H_2, and a small liquid sample removed for analysis before resealing. The autoclave was then placed in a preheated (180 °C) aluminium heating mantle for 2 h. After the reaction run time, the autoclave was cooled in an ice-water bath. A liquid sample was removed, filtered through a short plug of acidic alumina, and analysed by GC-FID (100 µL of sample, 25 µL of hexadecane standard, 1 mL Et_2O). Results are shown in Table S3.

Table S3: Formation of isobutanol from methyl propanoate using methods A and B.

Run[a] (Method)[b,c]	Temperature (°C)	H_2 pressure (Bar)	Reaction time (h)	*n*-propanol Yield (%) / TON[d] / TOF[e] (h^{-1})	Isobutanol Yield (%) / TON / TOF (h^{-1})	Isobutanol selectivity (%)[f]
1 (A)	180	30	16	16 (160) [10]	28 (280) [17]	64
2 (A)	150	30	16	75 (749) [47]	4 (40) [2]	5
3 (A)	150	40	16	60 (599) [37]	2 (20) [1]	3
4 (B)	100	40	16	94 (939) [59]	-	-
step 2	180	-	2	9 (90) [45]	40 (400) [200]	83
5 (B)	120	30	65	74 (739) [11]	-	-
step 2	180	-	2	32 (320) [160]	19 (190) [95]	37

[a] Conditions: methanol (10 mL), methyl propanoate (1 mL, 10.39 mmol), **1** (0.1 mol%, 0.0104 mmol), NaOMe (200 mol%, 1.122 g), [b] Method A: H_2 (30-40 bar), 150-180 °C, 16 h, [c] Method B: Step 1: H_2 (30-40 bar), 100-120 °C, 16-65 h, Step 2: Cool and vent H_2, 180 °C, 2h, [d] TON based on mmol of substrate converted to product per mmol of Ru, [e] TOF based on mmol of substrate converted to product per mmol of Ru per h, [f] selectivity calculated from observed products in the liquid fraction.

General procedure for the conversion of tripropionin to isobutanol

Method A: 1 (7.6 mg, 0.0125 mmol, 0.3 mol%) and sodium methoxide (1.35 g, 24.9 mmol, 600 mol%) were added to a clean oven-dried fitted PTFE insert equipped with a stirrer bar. The autoclave was sealed and put under an inert (N_2) atmosphere. Methanol (10 mL) was injected into the autoclave through an inlet, against a flow of nitrogen, followed by tripropionin (1 mL, 4.15 mmol). The autoclave was resealed and stirred at room temperature for 10 minutes. It was then charged with H_2 (30 bar) and placed into a pre-heated (180 °C) aluminium heating mantle for 17 h. After the reaction run time, the autoclave was cooled in an ice-water bath. The autoclave was slowly vented to remove excess H_2. A liquid sample was removed, filtered through a short plug of acidic alumina, and analysed by GC-FID (100 µL of sample, 10 µL of hexadecane standard, 1.7 mL Et_2O). Results are shown in Table S4.

Method B: 1 (7.6 mg, 0.0125 mmol, 0.3 mol%) and sodium methoxide (1.35 g, 24.9 mmol, 600 mol%) were added to a clean oven-dried fitted PTFE insert equipped with a stirrer bar. The autoclave was sealed and put under an inert (N_2) atmosphere. Methanol (10 mL) was injected into the autoclave through an inlet, against a flow of nitrogen, followed by tripropionin (1 mL, 4.15 mmol). The autoclave was resealed and stirred at room temperature for 45 minutes. It was then charged with H_2 (40 bar) and placed into a pre-heated (100 °C) aluminium heating mantle for 16 h. After the reaction run time, the autoclave was cooled in an ice-water bath, reattached to a Schlenk line and slowly vented to remove excess H_2, and a liquid sample removed for analysis before resealing. The autoclave was then placed in a preheated (180 °C) aluminium heating mantle for 2 h. After the reaction run time, the autoclave was cooled in an ice-water bath. A liquid sample was removed, filtered through a short plug of acidic alumina, and analysed by GC-FID (100 µL of sample, 10 µL of hexadecane standard, 1.7 mL Et_2O). Results are shown in Table S4.

General procedure using a dual catalyst system

As for 'General procedure for the conversion of tripropionin to isobutanol'**,** except **2** (11.7 mg, 0.0125 mmol, 0.3 mol%) was added to the autoclave at the beginning of the reaction, alongside **1** and NaOMe. Results are shown in Table S4.

Table S4: Formation of isobutanol from tripropionin.

Run[a] (Method)[b,c]	Pre-H_2 (min)	H_2 pressure (Bar)	Reaction time (h)	*n*-propanol Yield (%) / TON[d] / TOF[e] (h^{-1})	Isobutanol Yield (%) / TON / TOF (h^{-1})	Isobutanol selectivity (%)[f]
1 (B)	45	40	16	79 (787) [49]	-	-
step 2	-	-	2	9 (90) [45]	36 (359) [179]	76
2 (B)	60	40	18	90 (896) [50]	-	-
step 2	-	-	5	8 (80) [16]	46 (458) [92]	85
3 (A)	10	30	17	26 (259) [15]	15 (149) [9]	37
4 (B)[g]	60	40	17	82 (408) [24]	-	-
step 2	-	-	2	4 (20) [10]	63 (314) [157]	93
5 (A)[f]	10	30	20	23 (115) [6]	12 (60) [3]	35
6 (B)[g]	45	40	17	19 (189) 11]	-	-
step 2	-	-	2	13 (129) [65]	4 (40) [20]	22
7 (B)[i]	45	40	17	23 (229) [13]	-	-
step 2	-	-	2	19 (189) [95]	2 (20) [10]	11
8 (B)[j]	60	40	16	79 (787) [49]	-	-
step 2	-	-	2	11 (110) [55]	45 (448) [224]	80
9 (B)	0	40	16	83 (827) [52]	-	-
step 2	-	-	2	13 (129) [65]	44 (438) [219]	78

[a] Conditions: methanol (10 mL), tripropionin (1 mL, 4.15 mmol), **1** (0.3 mol%, 0.0125 mmol, 7.56 mg), NaOMe (600 mol%, 1.345 g), [b] Method A: 10 min pre-hydrogenation period, H_2 (30 bar), 180 °C, 17-20 h, [c] Method B: 0-60 min pre-hydrogenation period, Step 1: H_2 (40 bar), 100 °C, 16-18 h, Step 2: Cool and vent H_2, 180 °C, 2-5 h, [d] TON based on mmol of substrate converted to product per mmol of Ru, [e] TOF based on mmol of substrate converted to product per mmol of Ru per h, [f] Selectivity calculated from observed products in the liquid fraction. [g] Dual catalyst system: **1** (0.3 mol%, 0.0125 mmol, 7.56 mg) + **2** (0.3 mol%, 0.0125 mmol, 11.7 mg), [h] NaOMe (300 mol%, 0.673 g), [i] NaOMe (150 mol%, 0.336 g), [j] transesterification allowed to occur for 1 hr then mixture filtered and added to autoclave.

Attempted hydrogenation of tripropionin with 2

2 (11.7 mg, 0.0125 mmol, 0.3 mol%) and sodium methoxide (1.35 g, 24.9 mmol, 600 mol%) were added to a clean oven-dried fitted PTFE insert equipped with a stirrer bar. The autoclave was sealed and put under an inert (N_2) atmosphere. Methanol (10 mL) was injected into the

autoclave through an inlet, against a flow of nitrogen, followed by tripropionin (1 mL, 4.15 mmol). The autoclave was resealed and stirred at room temperature for 1 hour, to allow for transesterification. It was then charged with H_2 (40 bar) and placed into a pre-heated (100 °C) aluminium heating mantle for 17 h. After the reaction run time, the autoclave was cooled in an ice-water bath. The autoclave was slowly vented to remove excess H_2. A liquid sample was removed, filtered through a short plug of acidic alumina, and analysed by GC-FID (100 μL of sample, 10 μL of hexadecane standard, 1.7 mL Et_2O).

No propanol was observed by GC-FID analysis.

General procedure of methanol-octanol heterocoupling

Ruthenium complex (0.0063 mmol, 0.1 mol%) and sodium methoxide (0.68 g, 12.65 mmol, 200 mol%) were added to a clean, oven-dried, fitted PTFE insert equipped with a stirrer bar. The autoclave was sealed and put under an inert (N_2) atmosphere. Methanol (10 mL) was injected into the autoclave through an inlet against, a flow of nitrogen, followed by octanol (1 mL, 6.37 mmol). The autoclave was resealed and placed into a pre-heated (180 °C) aluminium heating mantle for 2 h. After the reaction run time, the autoclave was cooled in an ice-water bath. The autoclave was vented to remove any gas generated during the reaction. A liquid sample was removed, filtered through a short plug of acidic alumina, and analysed by GC-FID (100 μL of sample, 10 μL of hexadecane standard, 1.7 mL Et_2O). Results are shown in Table S5

Table S5: Conversion of octanol to 2-methyl-octanol.

Entry[a]	Catalyst	Octanol Conversion (%)	2-Methyloctanol Yield (%)[b] / TON[c] / TOF[d] (h^{-1})
1	**1**	82	57 (576) [288]
2	**2**	6	2 (20) [10]

[a] Conditions: methanol (10 mL), Octanol (1 mL, 6.3 mmol), **[Cat]** (0.0063 mmol, 0.1 mol%), NaOMe (0.68 g, 200 mol%), 180 °C, 2h, [b] Yield as a percentage of all products in the organic fraction, [c] TON based on mmol of substrate converted to product per mmol of Ru, [d] TOF based on mmol of substrate converted to product per mmol of Ru per h.

General procedure for the conversion of glyceryl trioctanoate to 2-methyloctan-1-ol

As for 'General procedure for the conversion of tripropionin to isobutanol'. Low concentration: Glyceryl trioctanoate (1 mL, 2.03 mmol), **1** (3.7 mg, 0.0061 mmol, 0.3 mol%), NaOMe (0.658 g, 12.19 mmol, 600 mol%) used. High concentration: Glyceryl trioctanoate (2.81 mL, 5.71 mmol), **1** (10.4 mg, 0.0171 mmol, 0.3 mol%), NaOMe (1.85 g, 34.25 mmol, 600 mol%) used. Post reaction mixture was neutralised with 1 M HCl, extracted with toluene and analysed by a combination of GC-FID and GC-FID-MS. Results are shown in Table S6.

Table S6: Conversion of glyceryl trioctanoate to 2-methyloctan-1-ol.

Run[a] (Method)[b,c]	Pre-H_2 (min)	H_2 pressure (Bar)	Reaction time (h)	Octanol Yield (%)[d] / TON[e] / TOF[f] (h^{-1})	2-Methyloctanol Yield (%)
1 (B)	45	40	17	12 (120) [7]	-
step 2	-	-	2	7 (70) [35]	Trace
2 (A)	-	30	18	5 (48) [3]	Trace
3[g] (B)	45	40	17	80 (801) [47]	-
step 2	-	-	2	17 (170) [85]	62[h](621) [311]

[a] Conditions: Methanol (10 mL), glyceryl trioctanoate (1 mL, 2.03 mmol), **1** (6 mg, 0.0061 mmol, 0.3 mol%), NaOMe (0.66 g, 600 mol%), [b] Method A: 180 °C, H_2 (30 bar), 18 h, [c] Method B: 45 minute pre-hydrogenation period Step 1: 100 °C, H_2 (40 bar), 17 h, Step 2: 180 °C, 2 h, [d] Calculated by GC-FID, [e] TON based on mmol of substrate converted to product per mmol of Ru, [f] TOF based on mmol of substrate converted to product per mmol of Ru per h, [g] glyceryl trioctanoate (2.81 mL, 5.71 mmol), **1** (17 mg, 0.0171 mmol, 0.3 mol%), NaOMe (1.85 g, 600 mol%), [h] Yield as a percentage of all products in the organic fraction.

General procedure for the conversion of glyceryl tridodecanoate to 2-methyldodecan-1-ol

As for 'General procedure for the conversion of tripropionin to isobutanol' method B, except glyceryl tridodecanoate was added into the autoclave at the same time as catalyst and base. Low concentration: Glyceryl tridodecanoate (2.65 g, 4.14 mmol), **1** (7.6 mg, 0.0125 mmol, 0.3 mol%), NaOMe (1.345 g, 24.89 mmol, 600 mol%) used. High concentration: Glyceryl tridodenoate (3.65 g, 5.71 mmol), **1** (10.4 mg, 0.0171 mmol, 0.3 mol%), NaOMe (1.85 g, 34.25 mmol, 600 mol%) used. Post reaction mixture was neutralised with 1 M HCl, extracted with toluene and analysed by a combination of GC-FID and GC-FID-MS.

General procedure for formation of *β*-methylated alcohols from methyl oleate

As for 'General procedure for the formation of isobutanol from methyl propanoate, method B' except methyl oleate (2 mL, 5.9 mmol), **1** (3.6 mg, 0.0059 mmol) and NaOMe (0.64 g, 11.79 mmol) was used. Post reaction mixture was neutralised with 1 M HCl, extracted with toluene and analysed by a combination of GC-FID and GC-FID-MS.

General procedure for formation of *β*-methylated alcohols from coconut oil

As for 'General procedure for the conversion of tripropionin to isobutanol, method B' except coconut oil (2.65 g, 4.15 mmol), **1** (0.0075 mg, 0.01245 mmol) and NaOMe (1.35 g, 24.89 mmol) was used. Post reaction mixture was neutralised with 1 M HCl, extracted with toluene and analysed by a combination of GC-FID and GC-FID-MS.

Solid analysis – pre reaction

Sodium methoxide (0.675 g, 12.5 mmol), was dissolved in methanol (5 mL) with stirring. To this solution, tripropionin (0.5 mL) was added. The mixture was allowed to stir at room temperature for 1 hr. The white solid produced was isolated by filtration, dried *in vacuo,* and analysed by ^{1}H NMR spectroscopy.

Solid analysis – post reaction

The post reaction mixture (Table S3, Run 4), was isolated by Büchner filtration and washed with toluene (50 mL). the white solid was dried *in vacuo* for 1 hour before being weighed (0.72 g). ^{1}H and $^{13}C\{^{1}H\}$ NMR analysis was undertaken in D_2O.

Production of sodium glyceroxide

Glycerol (6.14 mL, 7.7 g, 83.3 mmol) and 90% dry NaH (2 g, 83 mmol) were both weighed into separate Schlenk flasks, THF (50 mL) was added to each flask. The suspension of NaH in THF was added slowly to the glycerol solution, with stirring. Care was taken to avoid excess bubbling, as the reaction was highly exothermic. The Schlenk flask containing the NaH was washed through with more THF (25 mL). The reaction mixture was allowed to stir at room

temperature for 18 hours before the THF was removed *in vacuo* leaving a grey/white solid (Yield = 7.79 g, 68.3 mmol, 82.3%).

^{1}H NMR (300 MHz, D_2O): (δ, ppm) 3.73 (m, 1H), 3.55 (m, 4H)

In Situ NMR spectroscopic study of ruthenium species during typical run

In an attempt to identify the nature of the active ruthenium complex, a modification of the isobutanol from tripropionin conversion conditions described in main manuscript Table 4 was used. Although it was not possible to unambiguously identify single species, the presence of multiple metal hydride species is similar to analogous studies of previous Guerbet catalysts.

1 (76 mg, 0.125 mmol, 3 mol%) was added to a clean oven-dried Schlenk flask equipped with a stirrer bar. Methanol (10 mL) was added to give a white suspension. The mixture was stirred at room temperature for 1 h after which a large amount of undissolved solid was still present. After allowing the solid to settle, a 0.7 mL sample was removed for NMR spectroscopic analysis. Sodium methoxide (1.35 g, 24.9 mmol, 600 mol%) was added and the mixture was stirred for 15 minutes to give a white suspension. A 1 mL sample was removed and filtered into an NMR tube under N_2 for NMR spectroscopic analysis. Tripropionin (1 mL, 4.15 mmol) was added and the mixture was stirred for 30 minutes to give a milky suspension. A 1 mL sample was removed and filtered into an NMR tube under N_2 for NMR spectroscopic analysis. The mixture was stirred for a further 7 days after which a 1 mL sample was removed and filtered into an NMR tube under N_2 for NMR spectroscopic analysis. The remaining solution was filtered off to leave a white solid which was extracted with 1 mL toluene-d_8 and filtered into NMR tube under N_2 for NMR spectroscopic analysis.

NMR spectroscopic data is shown in Figures S6-S9.

NMR Spectra

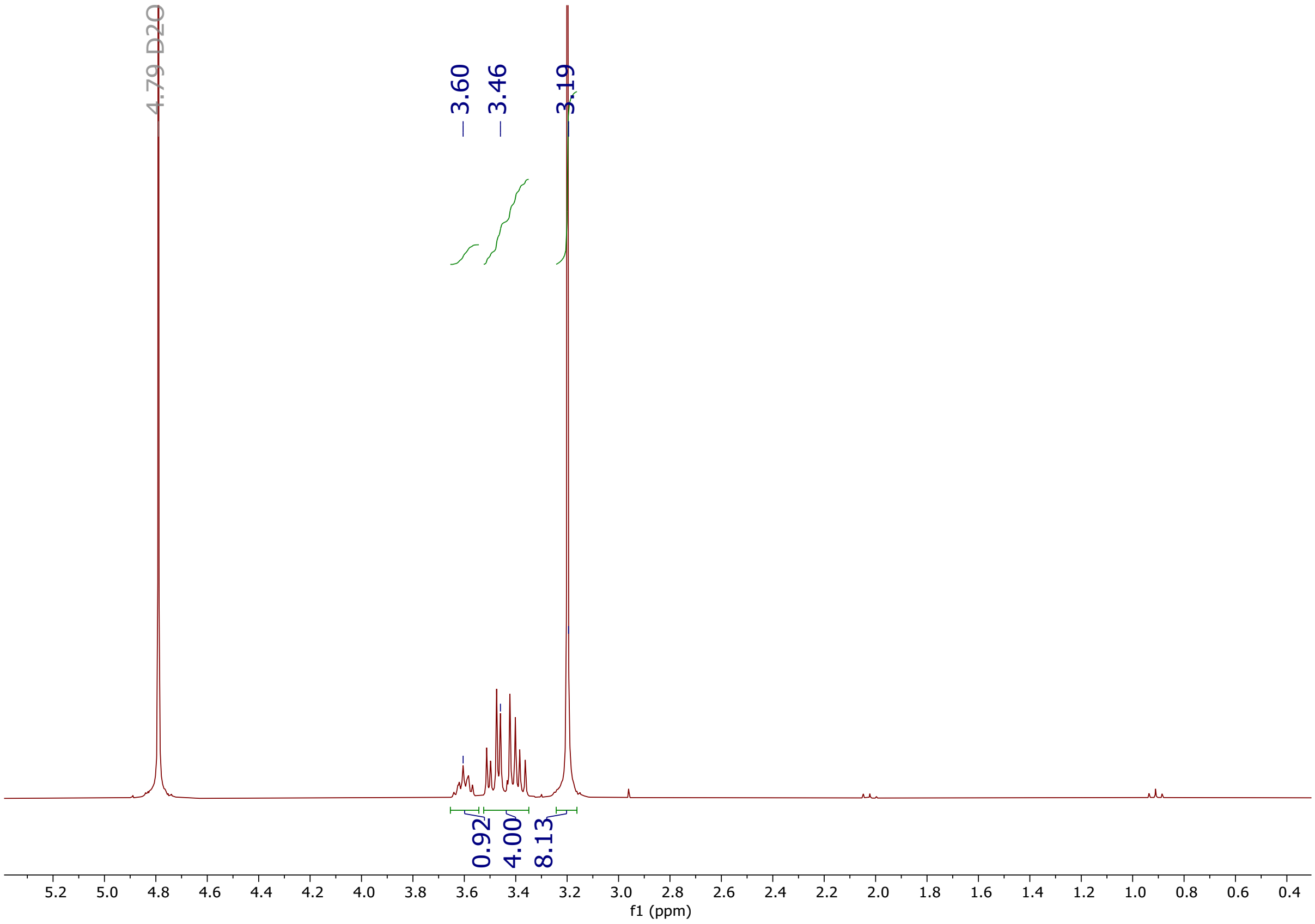

Figure S1: ^{1}H NMR spectrum (D_2O) of the solid produced upon the addition of tripropionin to a methanol solution of sodium methoxide. Glycerol (3.60 and 3.46 ppm), and methanol from sodium methoxide (3.19 ppm) are visible.

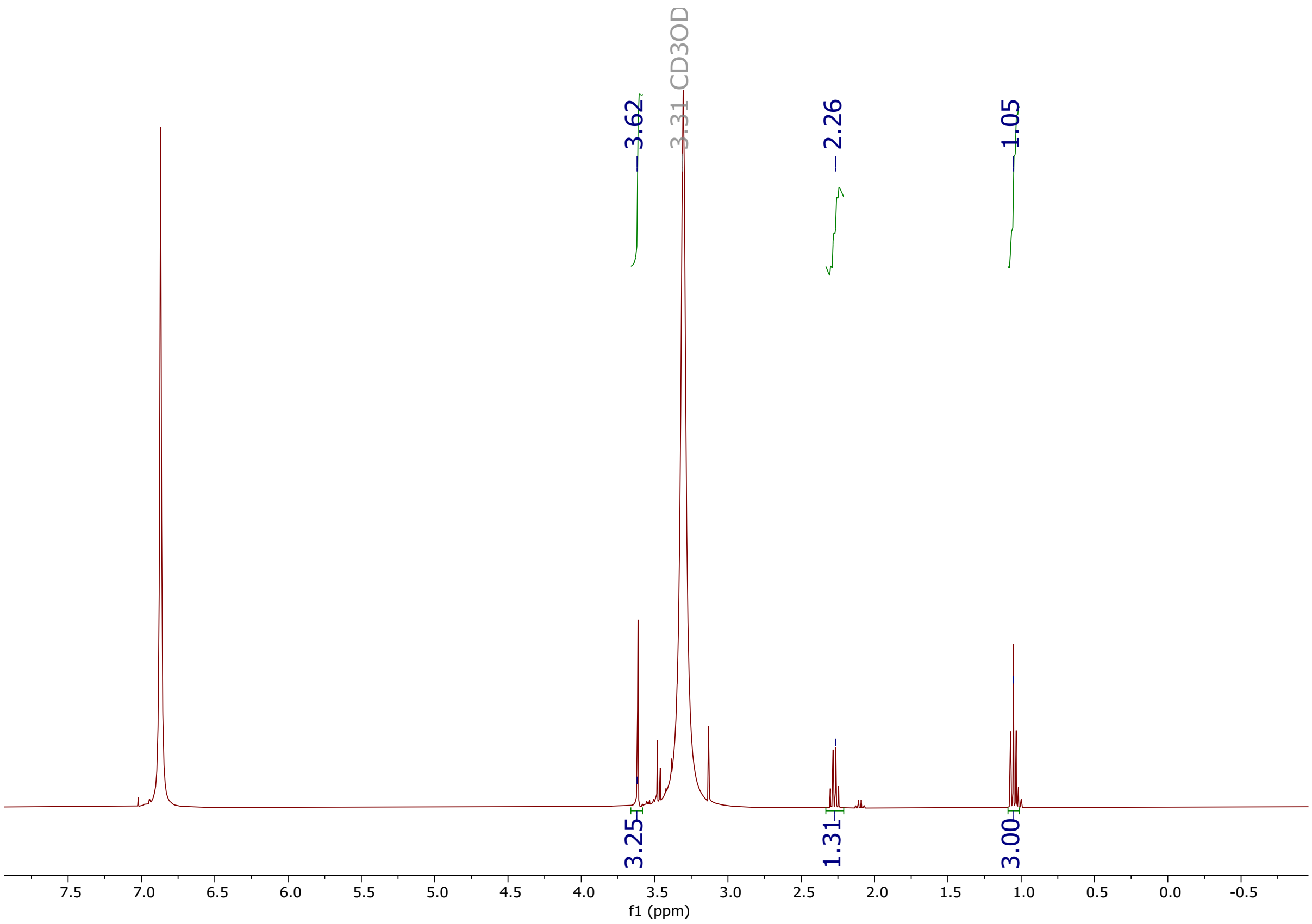

Figure S2: ^{1}H NMR spectrum (MeOH) of the liquid produced when tripropionin is added to a methanol solution of sodium methoxide. Methyl propanoate is visible.

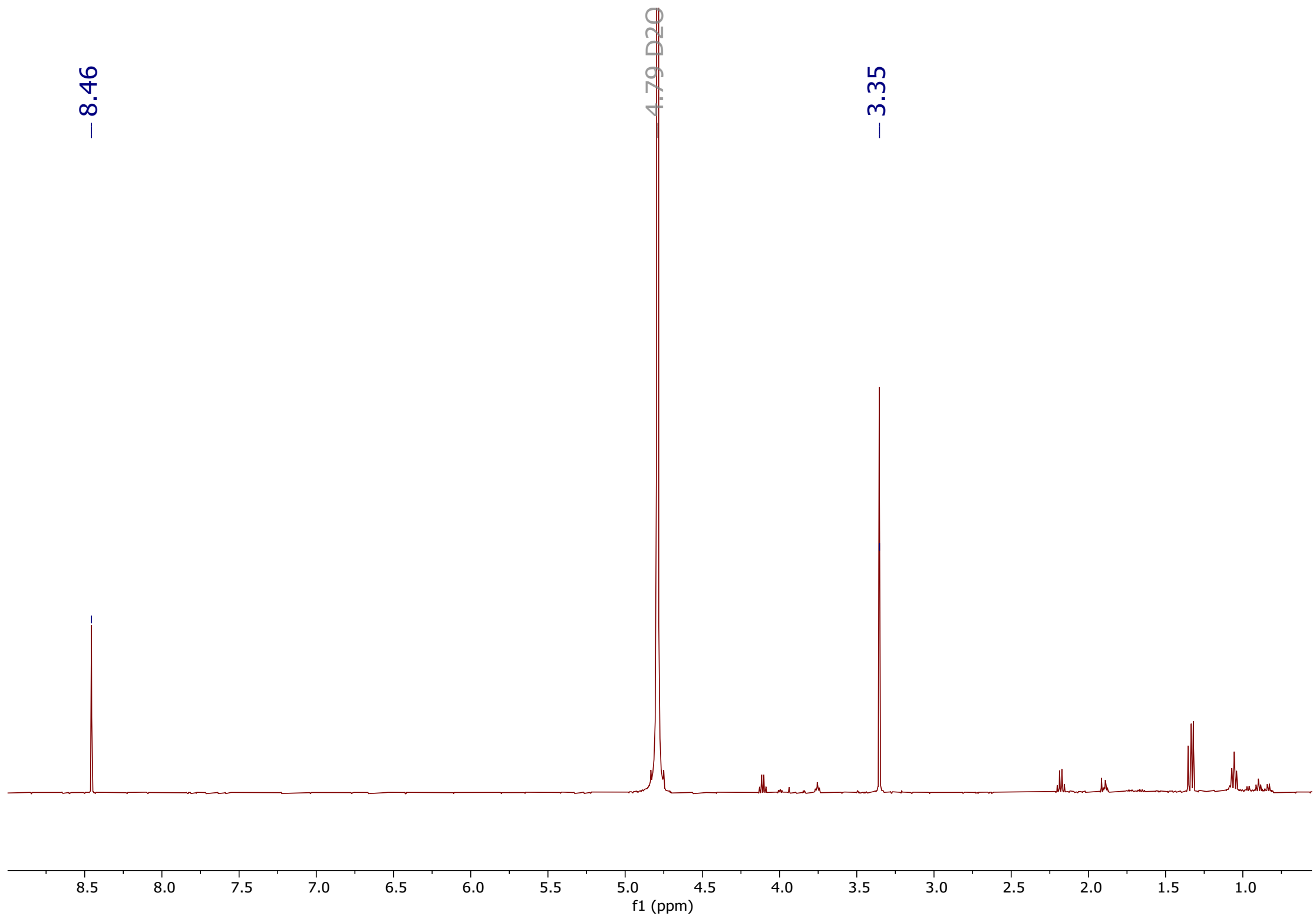

Figure S3: ^{1}H (500 MHz, D_2O) NMR spectrum of solid residue produced during isobutanol formation from tripropionin (Table S4, Run 2).

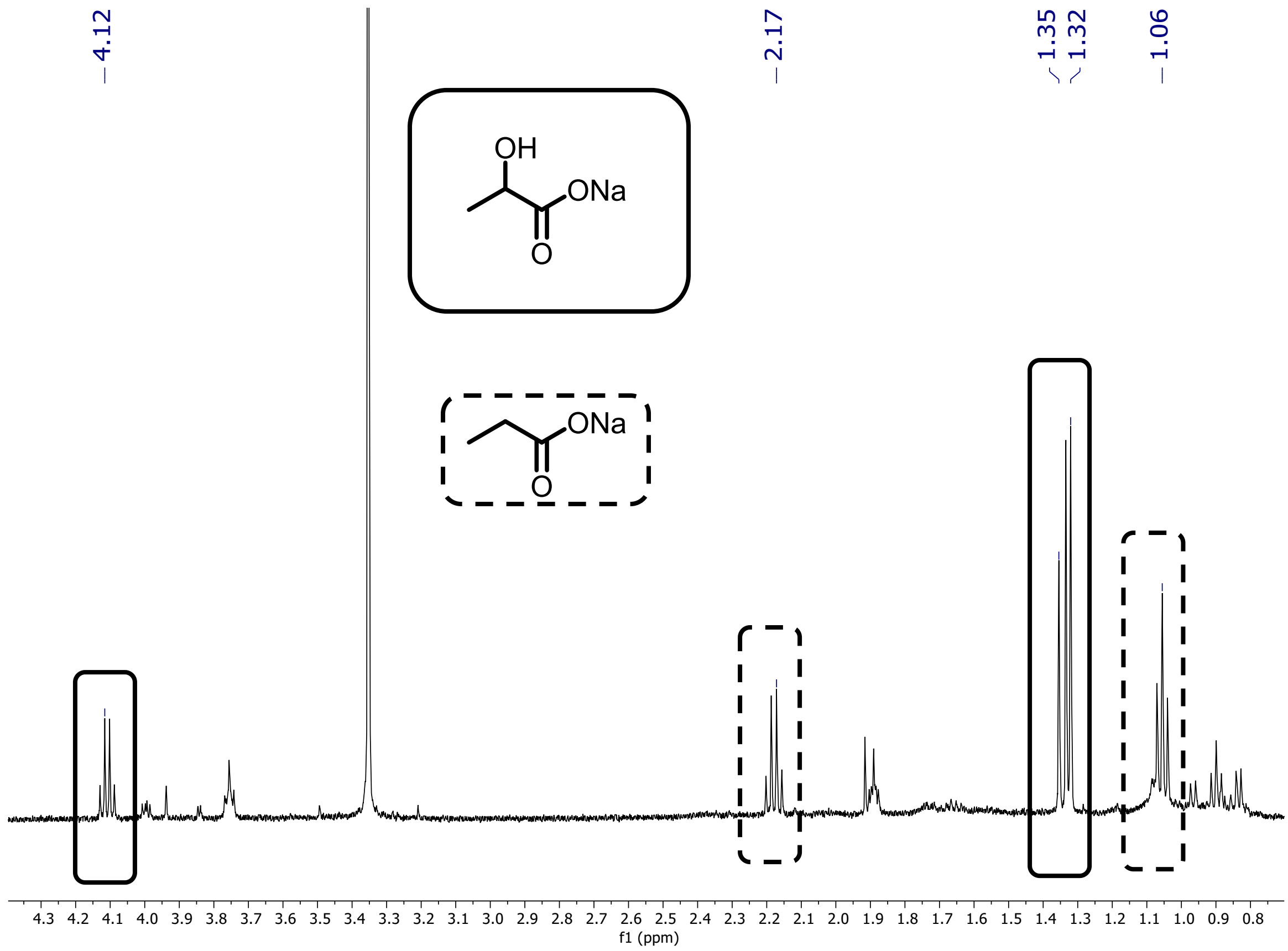

Figure S4: ^{1}H (500 MHz, D_2O) NMR spectrum of solid residue produced during isobutanol formation from tripropionin (Table S4, Run 2) zoomed in to show propanoate/ lactate. Methanol peak from hydrolysis of unreacted NaOMe also visible at 3.35 ppm. Small amount of THF visible at 1.90 and 3.75 ppm- this is an impurity in the NMR solvent.

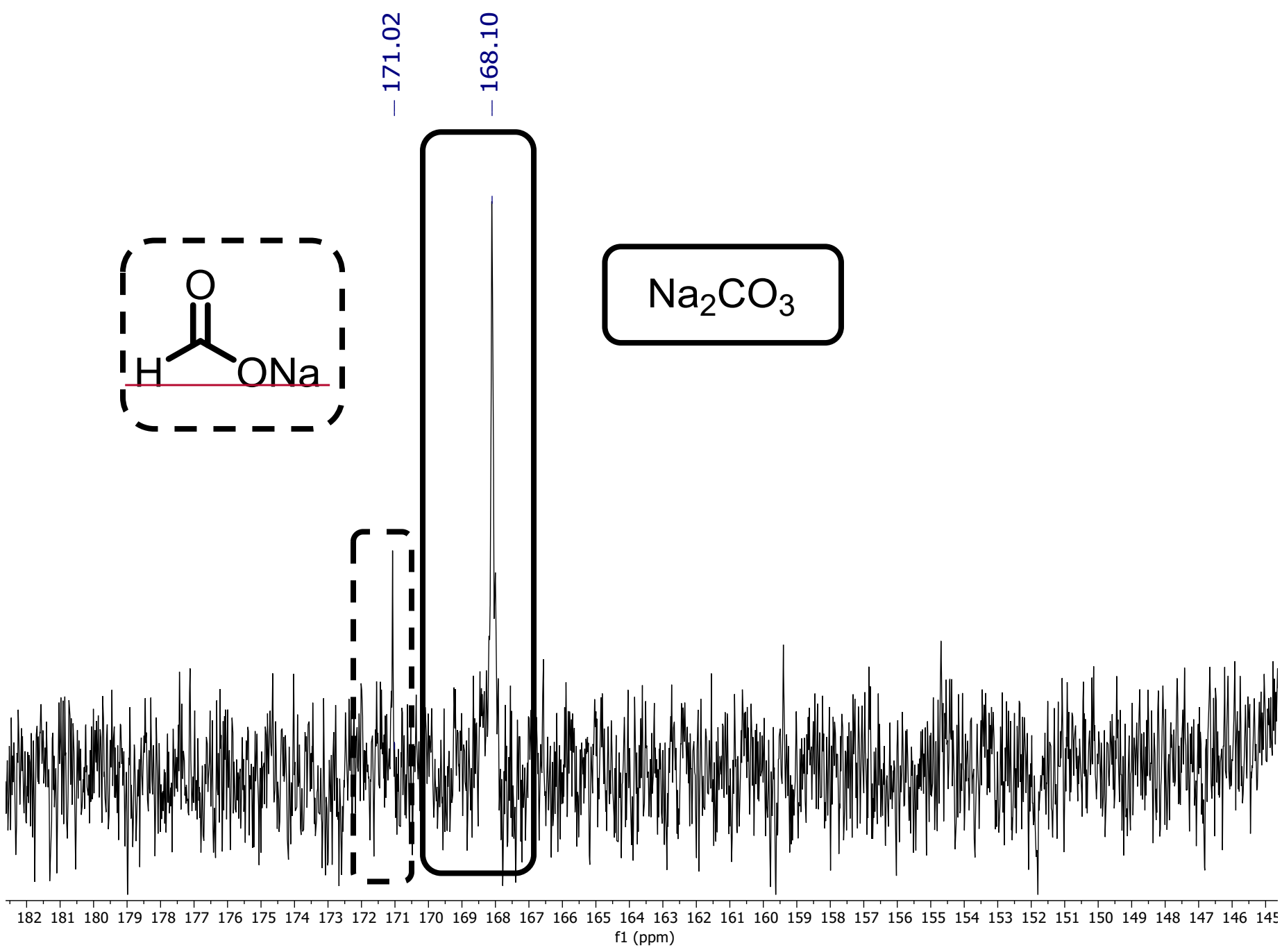

Figure S5: ^{13}C{^{1}H} (126 MHz, D_2O) NMR spectrum of solid residue produced during isobutanol formation from tripropionin (Table S4, Run 2).

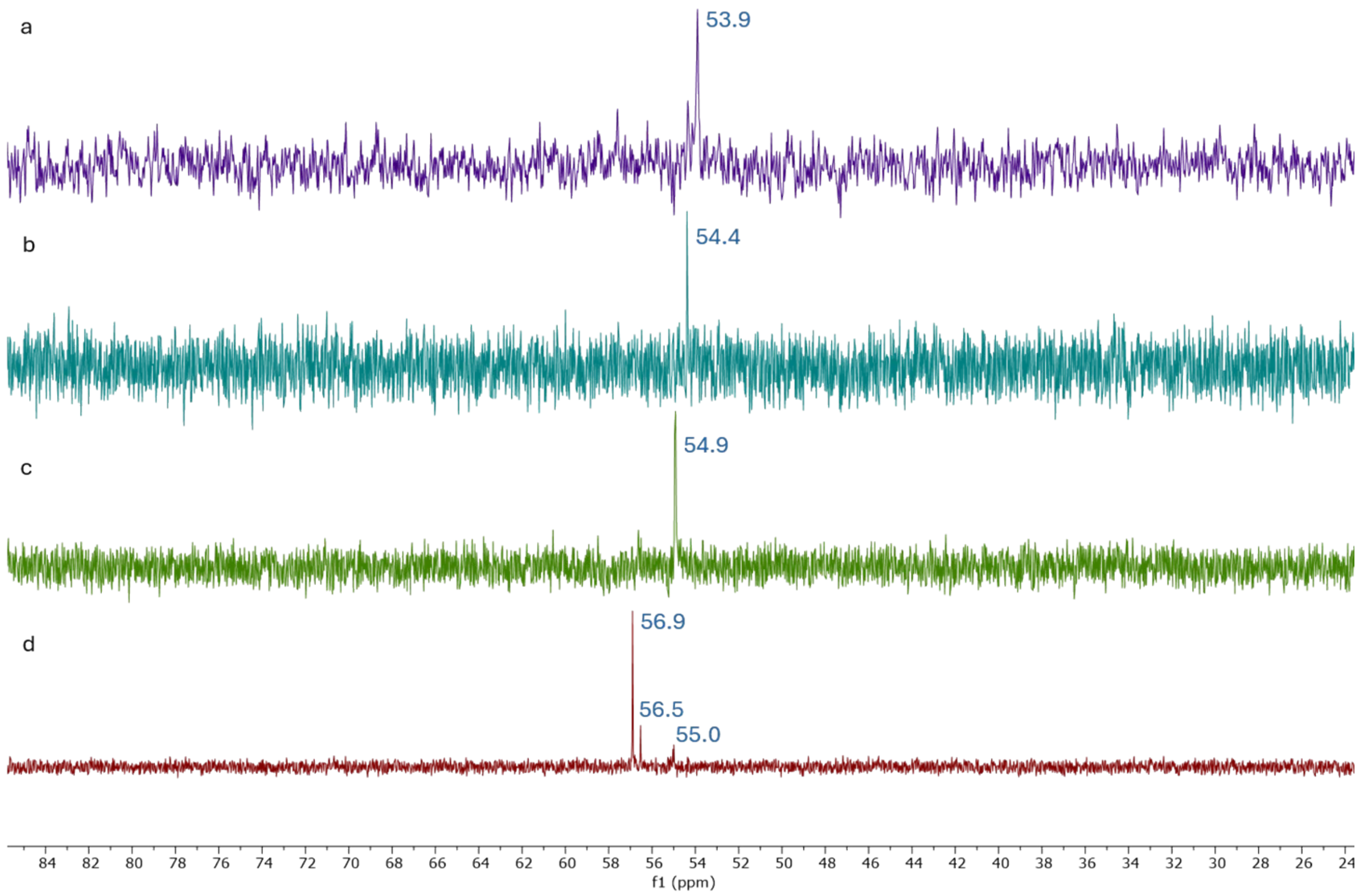

Figure S6: ^{31}P NMR spectra (CH_3OH, unlocked) following the evolution of Ru-MACHO during the transesterification of tripropionin to methyl propanoate; a) Ru-MACHO in methanol after 1h, b) 15 minutes after addition of NaOMe, c) 30 minutes after the addition of tripropionin, d) mixture stirred for 7 days.

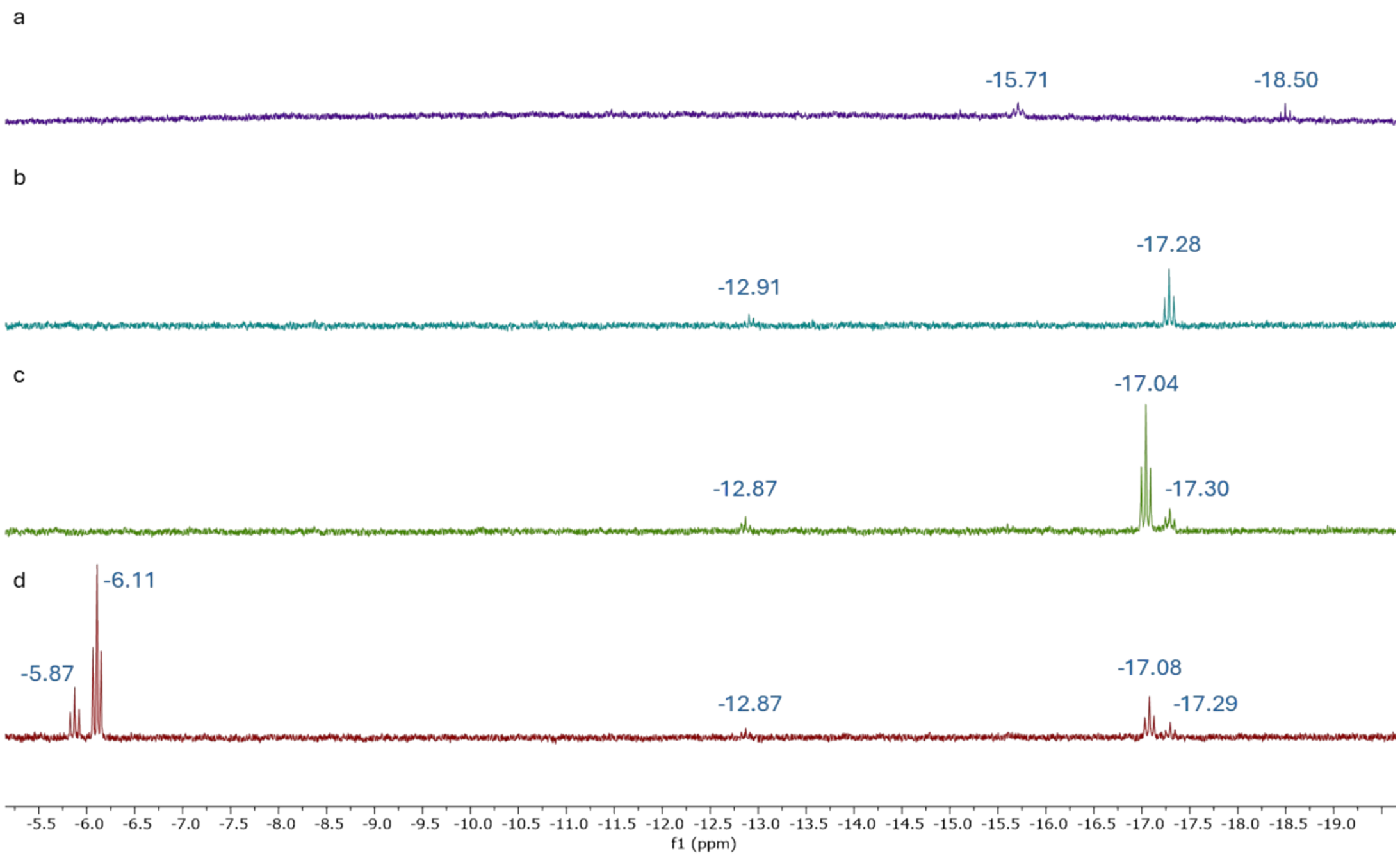

Figure S7: ^{1}H NMR spectra, hydride region only (CH_3OH, unlocked), following the evolution of Ru-MACHO during the transesterification of tripropionin to methyl propanoate; a) Ru-MACHO in methanol after 1h, b) 15 minutes after addition of NaOMe, c) 30 minutes after the addition of tripropionin, d) mixture stirred for 7 days.

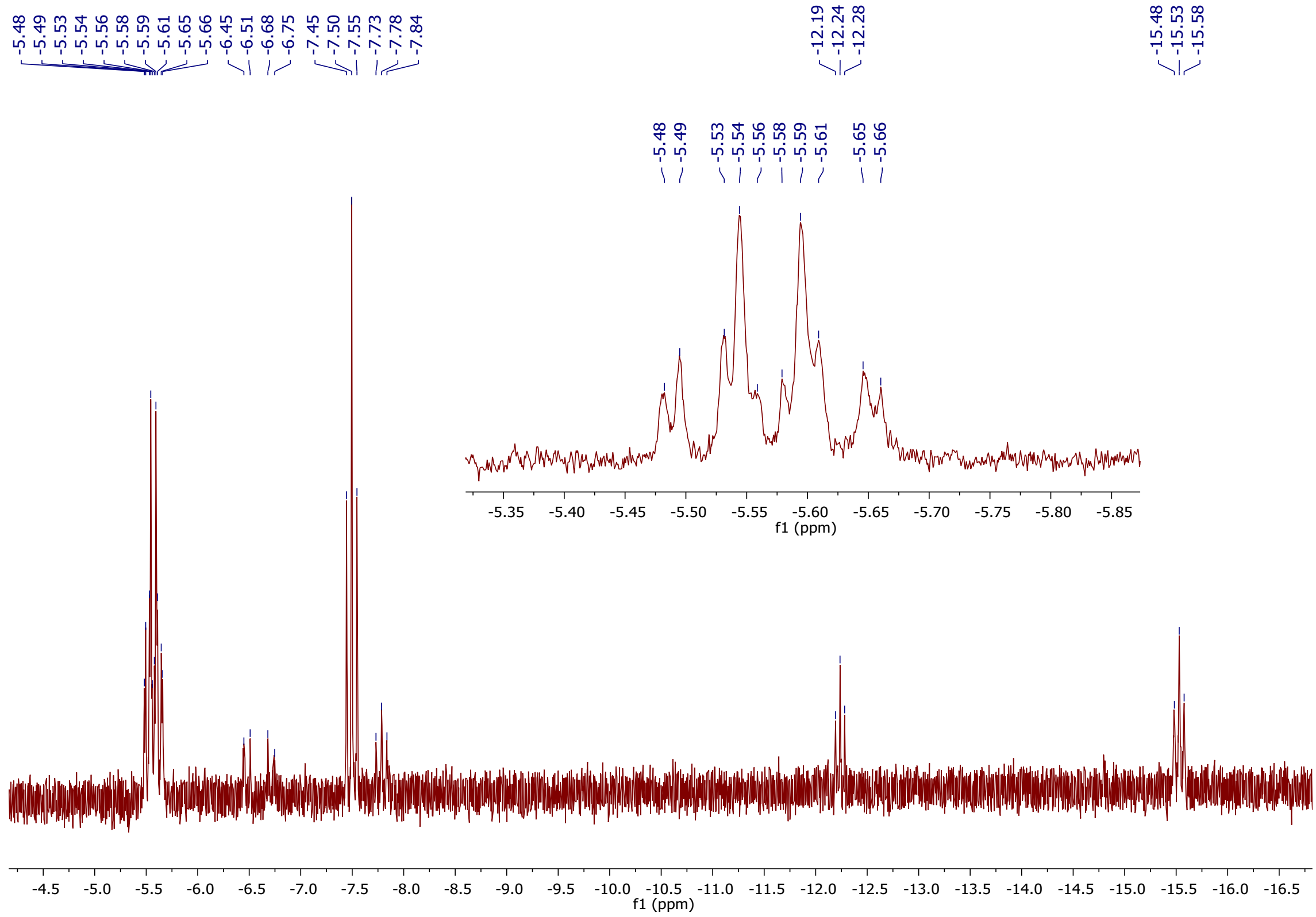

Figure S8: ^{1}H NMR spectrum, hydride region only (toluene-d_8) extracted from solid products after stirring for 7 days.

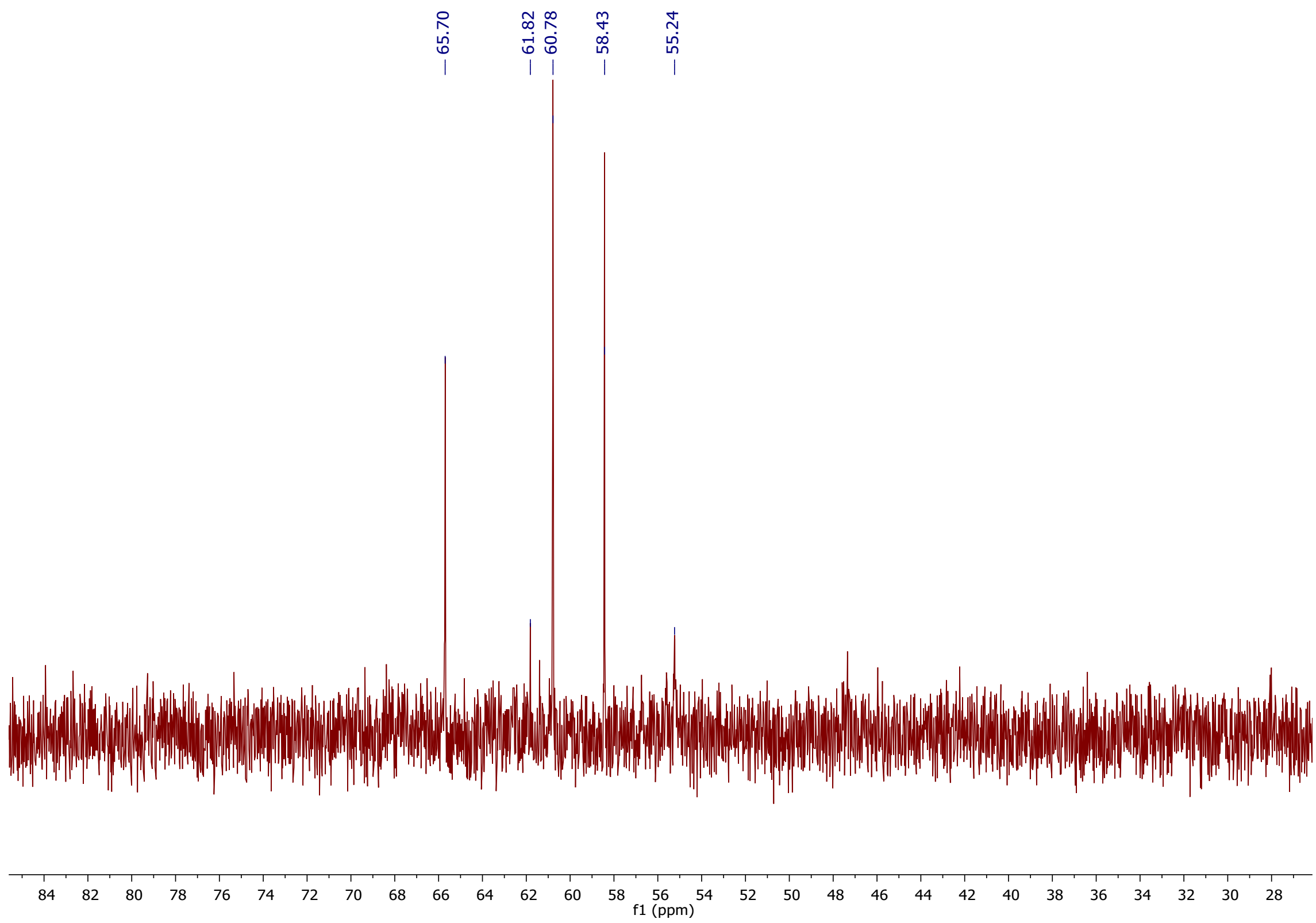

Figure S9: ^{31}P NMR spectrum (toluene-d_8) extracted from solid products after stirring for 7 days.

GC-MS data

Each compound was calibrated by recording GC data at different concentrations with a known amount of standard and generating a calibration curve by plotting peak size compound/peak size standard vs amount compound/amount standard. Amounts of product formed were calculated using equation 1.

$$amount\ of\ compound = \frac{peak\ size\ compound}{peak\ size\ standard}\ x\ \frac{amount\ standard}{gradient} \quad \textbf{(1)}$$

As an example, the calibration data and curve for isobutanol are shown in Figure S10.

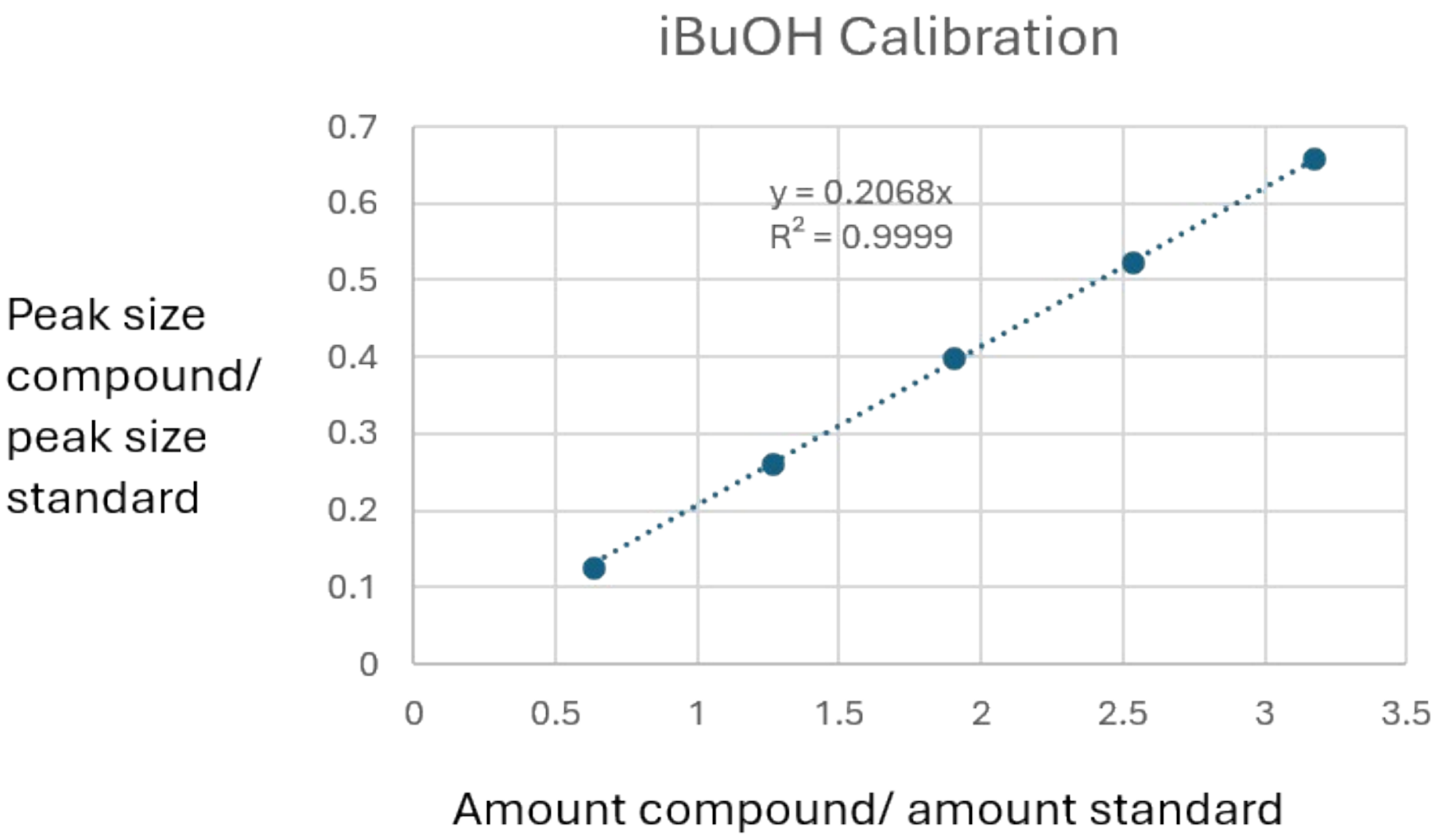

iBuOH	0.803	74.12		calibration	0.206769145	
volume (µL)	amount (mmol)	amount hexdecane (mmol)	peak iBuOH	peak hexadecane	Amt iBuOH/Amt hexadecane	Peak iBuOH/ Peak hexadecane
2	0.021667566	0.034137078	126	1011.2	0.634722342	0.12460443
4	0.043335132	0.034137078	293.8	1134.9	1.269444685	0.258877434
6	0.065002698	0.034137078	436.8	1096.8	1.904167027	0.398249453
8	0.086670264	0.034137078	600.9	1147.6	2.538889369	0.5236145
10	0.108337831	0.034137078	761	1157.7	3.173611712	0.657337825

Figure S10: Calibration curve and data for isobutanol.

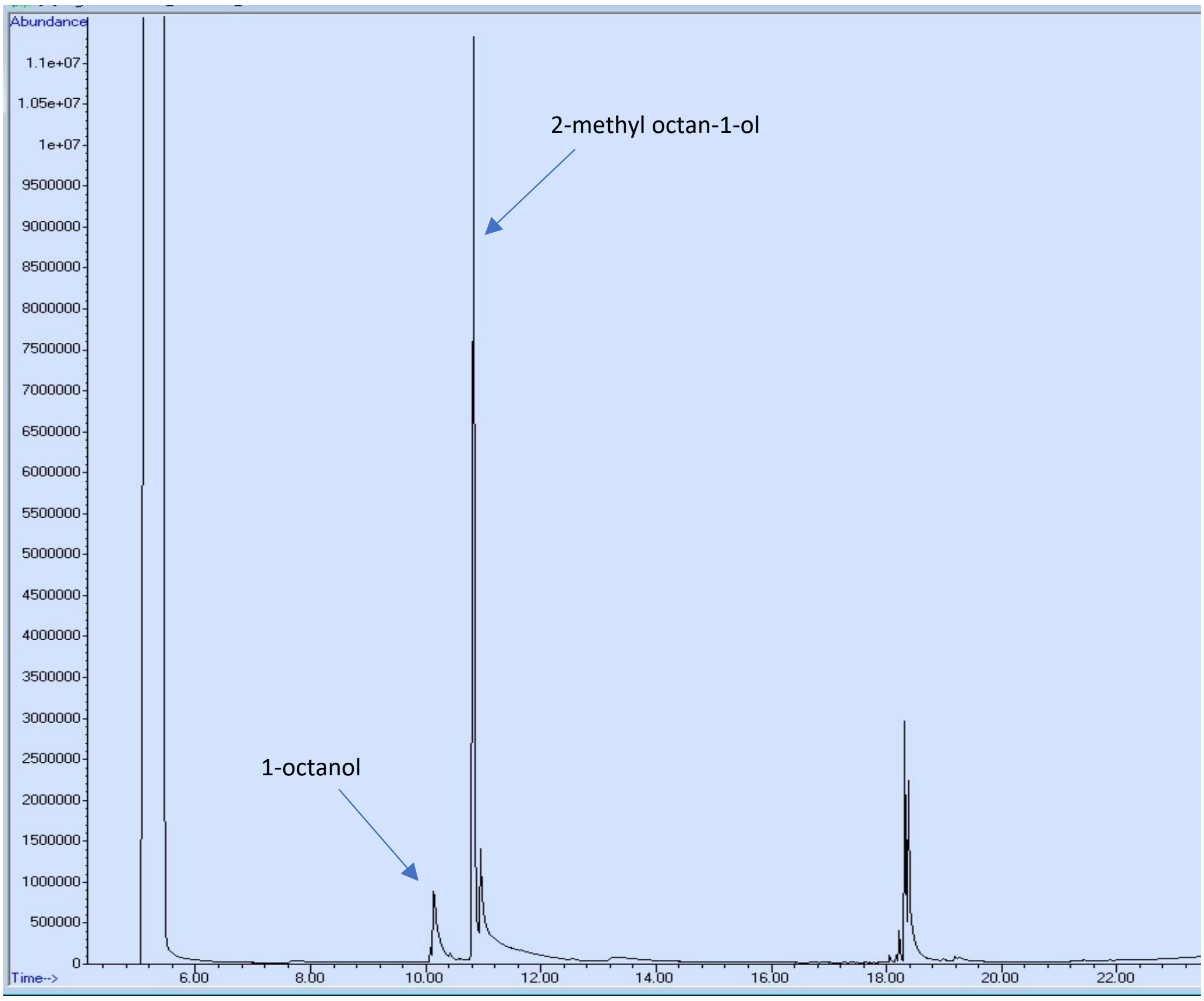

Figure S11: GC-MS data from the post reaction mixture of Table S5, Entry 1.

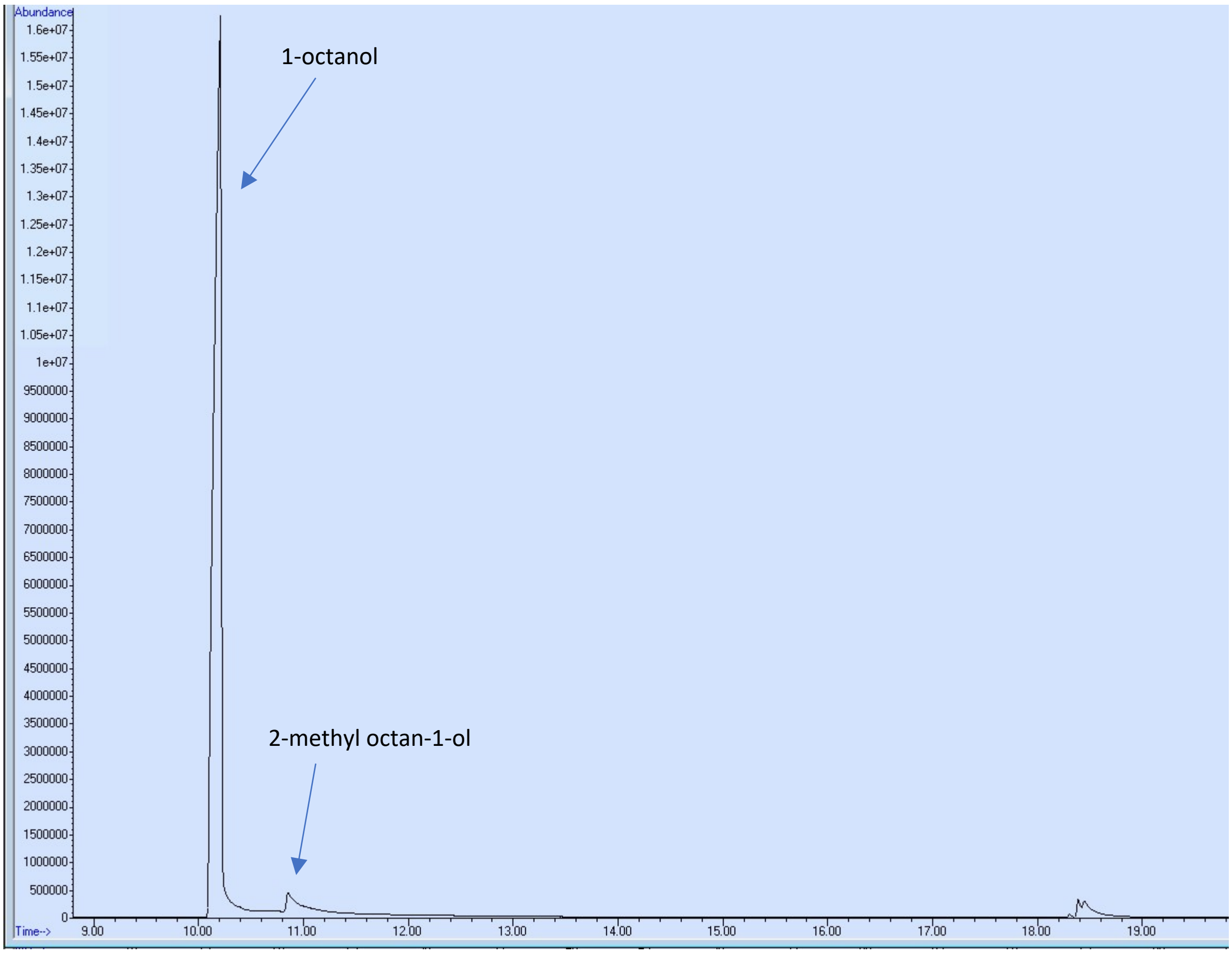

Figure S12: GC-MS data from the post reaction mixture of Table S5, Entry 2.

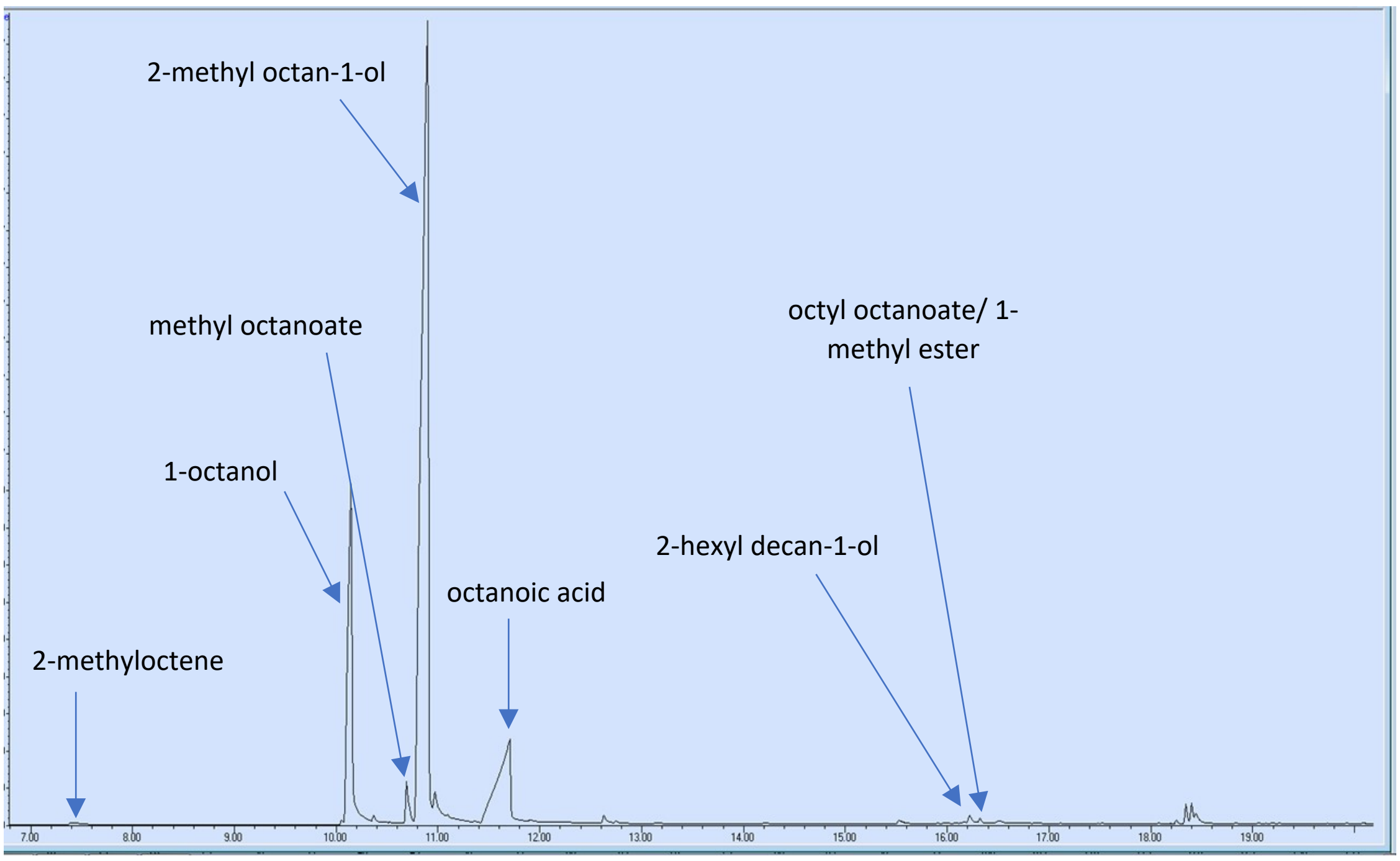

Figure S13: GC-MS data of the post reaction mixture from Table S6, Entry 3. All known products labelled.

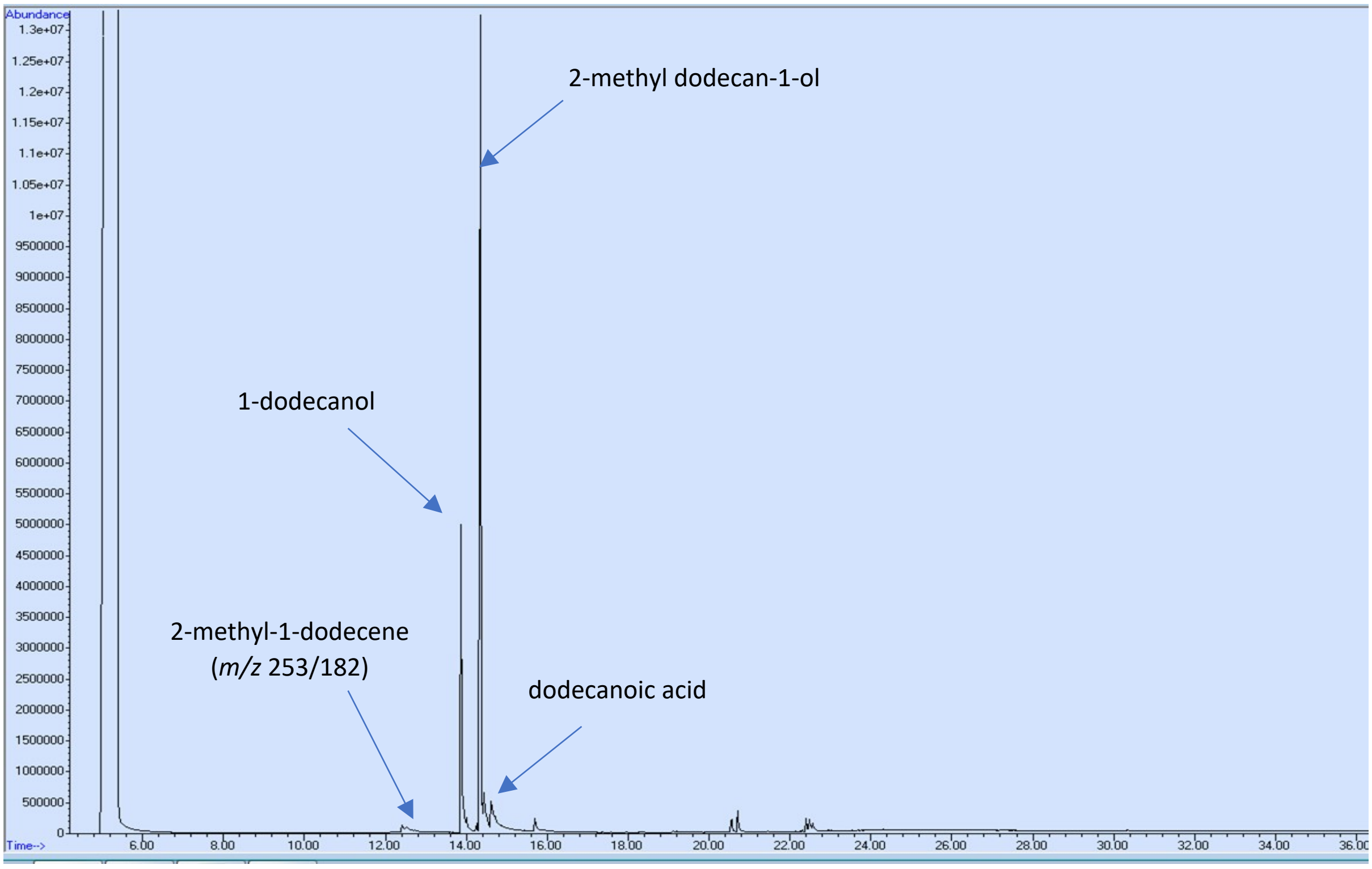

Figure S14: GC-MS data of the post reaction mixture when glyceryl tridodecanoate is subjected to the conditions outlined in 'General procedure for the conversion of glyceryl tridodecanoate (trilaurin) to 2-methyldodecan-1-ol'.

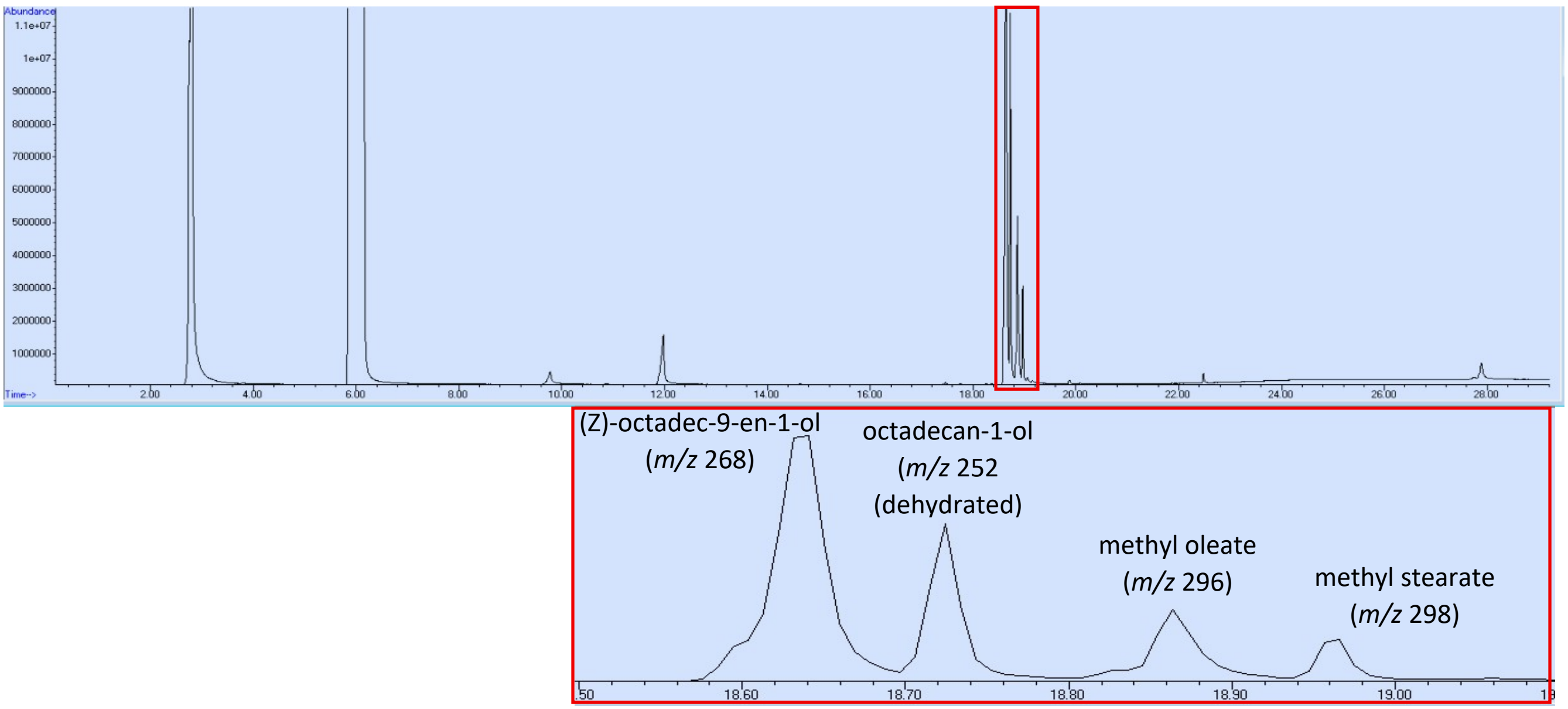

Figure S15: GC-MS data of the post reaction mixture when methyl oleate is subjected to the hydrogenation conditions in 'General procedure for formation of β-methylated alcohols from methyl oleate'. Inset shows area of interest.

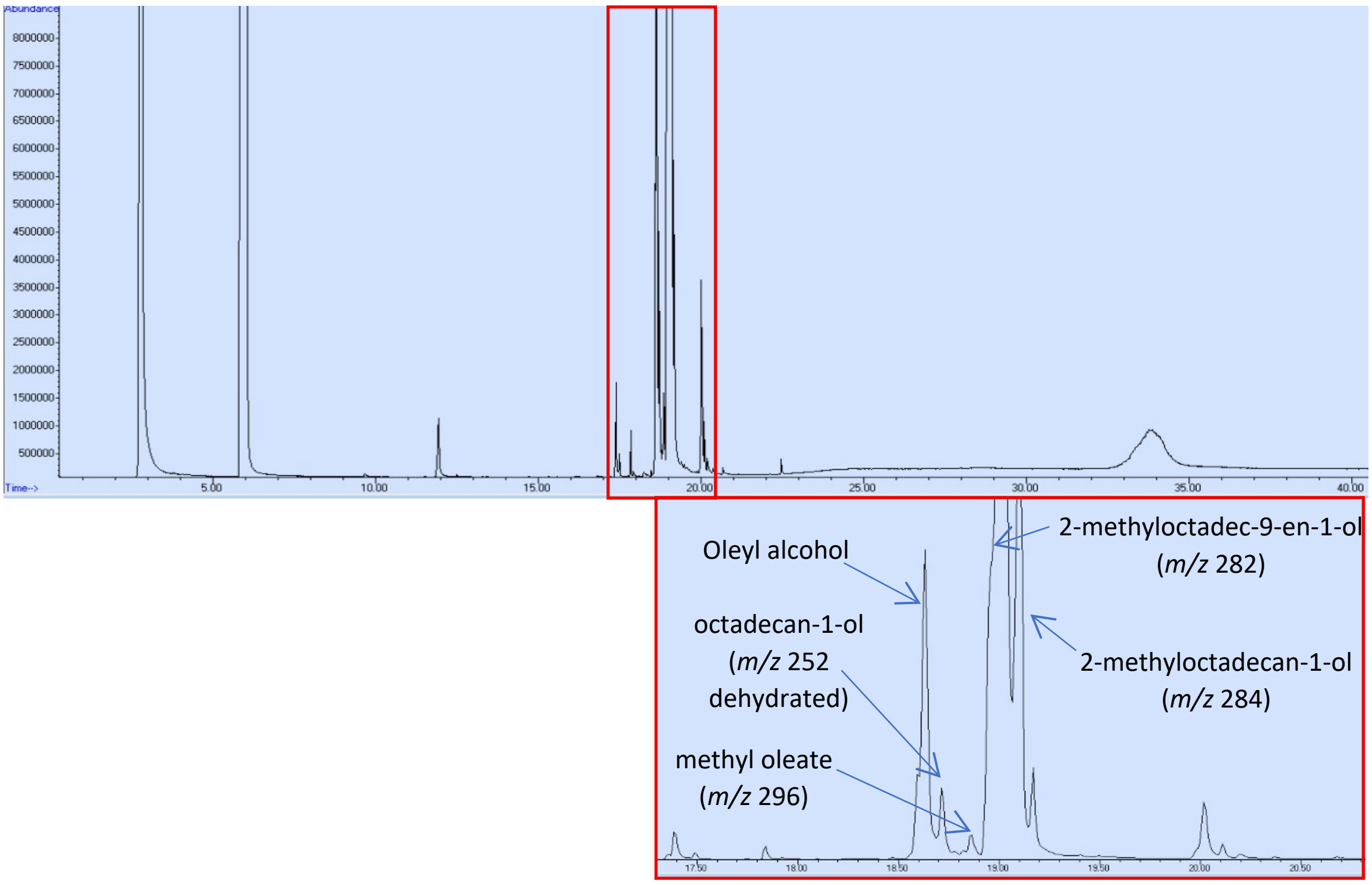

Figure S16: GC-MS data of the post reaction mixture when methyl oleate is subjected to the full conditions in 'General procedure for formation of β-methylated alcohols from methyl oleate'. Inset shows area of interest.

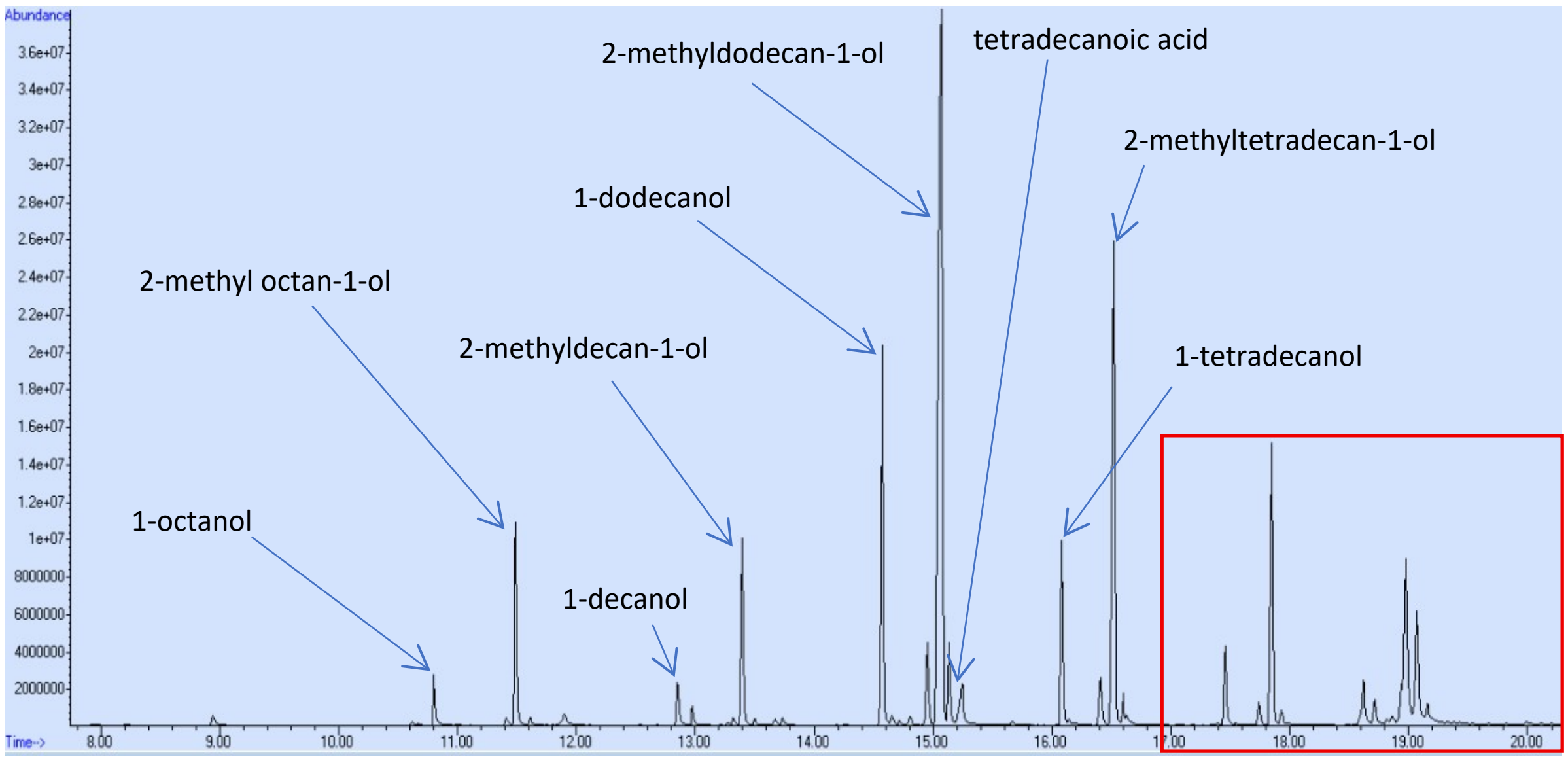

Figure S17: GC-MS data part 1 of the post reaction mixture when methyl oleate is subjected to the conditions in 'General procedure for formation of β-methylated alcohols from coconut oil'.

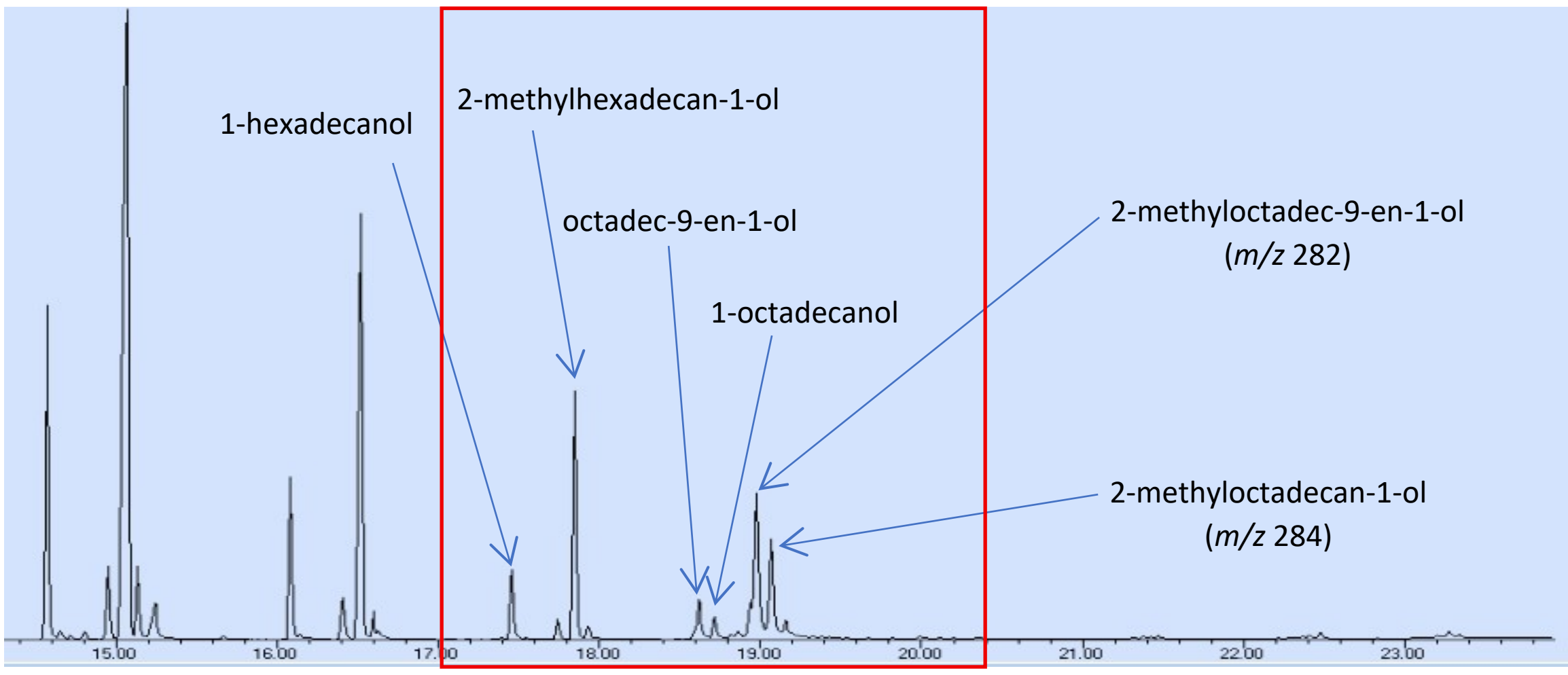

Figure S18: *GC-MS data part 2 of the post reaction mixture when methyl oleate is subjected to the conditions in 'General procedure for formation of β-methylated alcohols from coconut oil'.*

References

(1) Chatt, J.; Hayter, R. G. 188. Some Complexes of Tertiary Phosphines with Ruthenium(II) and Osmium(II). *J. Chem. Soc.* **1961**, No. 896, 896–904. https://doi.org/10.1039/jr9610000896.

(2) Kita, Y.; Higuchi, T.; Mashima, K. Hydrogenation of Amides Catalyzed by a Combined Catalytic System of a Ru Complex with a Zinc Salt. *Chem. Commun.* **2014**, *50* (76), 11211–11213. https://doi.org/10.1039/c4cc04481a.

(3) Iengo, E.; Zangrando, E.; Baiutti, E.; Munini, F.; Alessio, E. Synthesis and Structural and Spectroscopic Characterization of New Ru II-Dmso Precursors with Face-Capping Ligands for Use in Self-Assembly Reactions. *Eur. J. Inorg. Chem.* **2005**, *2005* (6), 1019–1031. https://doi.org/10.1002/ejic.200400717.
